# The Phytotoxin Myrigalone A Triggers a Phased Detoxification Programme and Inhibits *Lepidium sativum* Seed Germination via Multiple Mechanisms including Interference with Auxin Homeostasis

**DOI:** 10.3390/ijms23094618

**Published:** 2022-04-21

**Authors:** Kazumi Nakabayashi, Matthew Walker, Dianne Irwin, Jonathan Cohn, Stephanie M. Guida-English, Lucio Garcia, Iva Pavlović, Ondřej Novák, Danuše Tarkowská, Miroslav Strnad, Marta Pérez, Anne Seville, David Stock, Gerhard Leubner-Metzger

**Affiliations:** 1 Department of Biological Sciences, Royal Holloway University of London, Egham TW20 0EX, UK; kazumi.nakabayashi@rhul.ac.uk (K.N.); matthew.walker.2016@live.rhul.ac.uk (M.W.); marta.perez@rhul.ac.uk (M.P.); 2 Syngenta Jealott’s Hill International Research Centre, Bracknell RG42 6EY, UK; dianne.irwin@syngenta.com (D.I.); anne.seville@syngenta.com (A.S.); 3 Syngenta Crop Protection, LLC, Research Triangle Park, NC 27709, USA; josh.cohn@syngenta.com (J.C.); lucio.garcia@syngenta.com (L.G.); 4 National Center for Genome Resources, 2935 Rodeo Park Dr E, Santa Fe, NM 87505, USA; stephanie.english@syngenta.com; 5 Laboratory of Growth Regulators, Institute of Experimental Botany, Czech Academy of Sciences and Faculty of Science, Palacký University Olomouc, CZ-78371 Olomouc, Czech Republic; iva.pavlovic@upol.cz (I.P.); ondrej.novak@upol.cz (O.N.); tarkowska@ueb.cas.cz (D.T.); miroslav.strnad@upol.cz (M.S.)

**Keywords:** allelochemical and allelopathy, aquaporin-mediated water transport, ATP-binding cassette (ABC) transporter, auxin transport and homeostasis, gibberellin metabolism, *cis*-(+)-12-oxophytodienoic acid (OPDA) reductase, PIN auxin efflux carrier, WRKY transcription factors, seed germination, phytotoxin detoxification programme

## Abstract

Molecular responses of plants to natural phytotoxins comprise more general and compound-specific mechanisms. How phytotoxic chalcones and other flavonoids inhibit seedling growth was widely studied, but how they interfere with seed germination is largely unknown. The dihydrochalcone and putative allelochemical myrigalone A (MyA) inhibits seed germination and seedling growth. Transcriptome (RNAseq) and hormone analyses of *Lepidium sativum* seed responses to MyA were compared to other bioactive and inactive compounds. MyA treatment of imbibed seeds triggered the phased induction of a detoxification programme, altered gibberellin, *cis*-(+)-12-oxophytodienoic acid and jasmonate metabolism, and affected the expression of hormone transporter genes. The MyA-mediated inhibition involved interference with the antioxidant system, oxidative signalling, aquaporins and water uptake, but not uncoupling of oxidative phosphorylation or p-hydroxyphenylpyruvate dioxygenase expression/activity. MyA specifically affected the expression of auxin-related signalling genes, and various transporter genes, including for auxin transport (PIN7, ABCG37, ABCG4, WAT1). Responses to auxin-specific inhibitors further supported the conclusion that MyA interferes with auxin homeostasis during seed germination. Comparative analysis of MyA and other phytotoxins revealed differences in the specific regulatory mechanisms and auxin transporter genes targeted to interfere with auxin homestasis. We conclude that MyA exerts its phytotoxic activity by multiple auxin-dependent and independent molecular mechanisms.

## 1. Introduction

Surviving seed germination and seedling growth as the early stages of plant establishment in a natural or agricultural ecosystem, is a “first off the mark” challenge that requires responding successfully to biotic and abiotic environmental stressors [1,2,3]. This includes responding to phytotoxic chemicals interfering with germination and growth by mounting detoxification programmes and resistance mechanisms. Plant-derived natural phytotoxins include allelochemicals leached by “donor” plants into the rhizosphere to inhibit germination and growth of surrounding “target” plants [2,4,5,6,7]. Xenobiotics are mostly synthetic chemical substances that are not normally present in the environment and include the explosive 2,4,6-trinitrotoluene (TNT) [8], herbicides that act phytotoxic to target weeds [9,10,11,12] and herbicide safeners used to elicit detoxification programmes in crops [13]. There is an interest in the molecular targets of phytotoxic phytochemicals such as allelochemicals, as this may lead to templates for new classes of herbicides with new modes of action [14,15,16,17]. Very little is known about the molecular mechanisms, least of all mode(s) of action, underpinning the phytotoxicity of potential allelochemicals.

Transcriptome and hormone analyses are useful tools to obtain insight into the molecular responses triggered by a compound that inhibits seed germination and seedling growth. This approach alone is unlikely to lead to the identification of the phytotoxin’s primary molecular target site(s), but it will provide insight into the triggered gene expression response of the stress- and detoxification-related programmes, as well as into more specific hormonal, signalling and other biochemical pathways [4,18,19,20]. In contrast to the known commercial herbicides, which appear to have a single molecular target, natural phytotoxins often have multiple molecular target sites of different relative importance [14]. They are not necessarily reflected in the transcriptome changes triggered by the phytotoxin as they, in most cases, involve binding to specific proteins to interfere with their function as enzymes, transporters or signalling components. To what extent different groups of potential allelochemicals differ in the triggered stress- and detoxification-related programmes have not been compared in detail. Cross-comparisons of transcriptome datasets, specific bioactivities, and phytotoxicity phenotypes for responses to different phenylpropanoids, including coumarins [21,22,23], benzoxazinoids [4], chalcones and other flavonoids [16,19,23,24,25], terpenoids [7,18,26,27], alkaloids [6,20,23,28,29,30], and xenobiotics [8,13,31], may reveal more general and more specific molecular mechanisms and bioactivities.

Natural chalcones and their derivatives have numerous bioactivities and molecular targets of interest in pharmaceutical [32] and agrochemical [12,16,33] research. Myrigalone A (MyA) is a flavonoid, a rare C-methylated dihydrochalcone, in fruit leachates of *Myrica gale* (“sweet gale”, “bog myrtle”, Myricaceae), which is a deciduous shrub adapted to flood-prone habitats. Fruits secrete resin droplets of essential oils which contain MyA and other dihydrochalcones and chalcones [17,34,35,36]. The natural phytotoxin and putative allelochemical MyA inhibit seed germination and seedling growth [17,37,38]. By using *Lepidium sativum* (garden cress, Brassicaceae) as the target species, these works demonstrated that MyA enhanced testa (seed coat) permeability and early water uptake during the early phase of seed germination. During late germination, MyA inhibits micropylar endosperm (CAP) weakening and embryo growth, both processes required for the completion of germination by endosperm rupture and radicle protrusion [1,39,40]. The endosperm is a mediator of communication between the embryo and its environment, and it is therefore not surprising that abiotic (e.g., temperature) and biotic (e.g., allelochemicals) factors exert their germination-inhibiting effects, at least in part, by inhibiting CAP weakening. MyA still allowed germination, but increased the incidence of atypical endosperm rupture, inhibited endoreduplication in the radicle-hypocotyl growth zone (RAD), and interfered with cell expansion required for embryo growth [37]. Subsequent seedling root and shoot growth of *L. sativum* and other species were also inhibited by MyA [17]. The molecular mechanisms underpinning these bioactivities and the MyA-triggered stress-, detoxification- and hormone-related programmes are largely unknown. To address this, we conducted transcriptome and hormone analyses and compared these with other phytotoxins to identify more general and MyA-specific putative mechanisms.

## 2. Results and Discussion

### 2.1. Chalcones Differ in Their Phytotoxic Bioactivity and Inhibitory Action on Seed Germination

**Figure 1:**
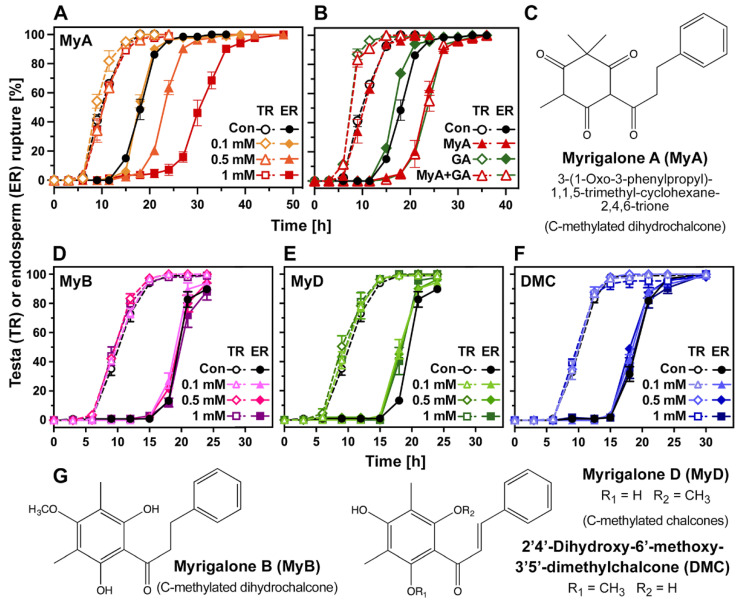
The effects of myrigalone A (MyA) and other dihydrochalcones and chalcones on *Lepidium sativum* seed germination. (**A**) The kinetics of testa rupture (TR) and subsequent endosperm rupture (ER) without (Con, control) or with MyA added at the concentrations indicated. Note that MyA inhibits ER but does not affect the timing of TR. (**B**) The effect of gibberellin (100 µM GA_4+7_) on the inhibitory action of 0.5 mM MyA. (**C**) Chemical structure of the dihydrochalcone MyA. (**D**–**F**) Germination kinetics of cress seeds in the presence of three other myrigalone analoga. (**G**) Chemical structure of the MyB, MyD and DMC. Seeds were incubated at 20 °C in continuous white light. Mean ± SEM values for three replicates, each with ca. 30 seeds, are shown.

Figure 1 shows that the dihydrochalcone myrigalone A (MyA) inhibits *Lepidium sativum* (cress) seed germination, while other dihydrochalcones (MyB) and chalcones (MyD, DMC) extracted from *M. gale* were completely inactive in the cress germination assay. Cress is a “classical target species” in seed germination and seedling growth assays for analysing phytotoxic, allelochemical and herbicidal activity of various compounds [17,23,24,27,37]. The advantages of the cress system for investigating chemicals include a clear distinction between testa rupture (TR) and endosperm rupture (ER) as successive visible events during seed germination, as well as emerged seedlings which allow a clear distinction between root and shoot growth [17,38,40]. In agreement with earlier work [37,38], the MyA-mediated inhibition of cress seed germination did not affect TR, but specifically delayed the subsequent ER in a dose-dependent manner (Figure 1A). MyA has also been shown to inhibit seedling root and shoot growth of cress and other species in a dose-dependent manner [17]. In contrast to MyA, six other dihydrochalcones and chalcones (Figure 1 and Figure S1D), the flavanone naringenin (Figure S1D) and the flavone acacetin (Figure S1E), all did not affect cress seed germination. The flavanone heliannone B from sunflower [24] and three flavones [23] are also known to inhibit cress germination and seedling growth. Comparative seed and seedling bioassays of trans-chalcone and derivatives with several crop species, their associated weeds, and the model *Arabidopsis thaliana*, revealed structure-activity relationships [16,25]. These studies also revealed that species differ in their responses to a specific compound and that germination and seedling growth responses do not necessarily coincide.

As for the myrigalones (Figure 1) and other flavonoids, coumarins [22] also differed in their bioactivity in the cress system. While daphnetin and psoralen did not affect cress seed germination (Figure S1E), the furanocoumarin angelicin delayed both TR and ER (Figure S1A). Treatment with the gibberellin (GA) biosynthesis inhibitor paclobutrazol also delayed both TR and ER (Figure S1C). This germination delay by paclobutrazol was fully reverted by GA (100 µM GA_4_). In contrast to this, GA treatment did not revert the inhibitory effects of 0.5 mM MyA (Figure 1B) or 0.1 mM angelicin (Figure S1B). It is known from earlier work using the inhibitor fluridone that MyA does not confer its inhibitory effect on seed germination through enhanced abscisic acid (ABA) biosynthesis [37,38]. Various phenylpropanoids also differ in their bioactivity; examples of this include *cis*-cinnamic acid, which is phytotoxic to seedling growth through interference with auxin transport and ferulic acid, which interferes with hormone homeostasis and auxin signalling [21]. Ferulic acid, however, did not affect cress seed germination (Figure S1E). A comparison of MyA with different inactive chalcones (Figure 1 and Figure S1) and other phytochemicals and xenobiotics (see Figures S1 and S2 for their chemical structures) is therefore suited to reveal more common as well as MyA-specific molecular mechanisms. To gain further insight into the molecular processes underpinning MyA’s inhibitory action on cress ER, we conducted hormone and transcriptome profiling, and compared the responses of identified differentially expressed genes to inactive myrigalones (MyB, MyD), to other active phytotoxic compounds, including angelicin (Figures S1 and S2).

### 2.2. MyA-Induced Hormone and Transcriptome Changes in Germinating Seeds

**Figure 2:**
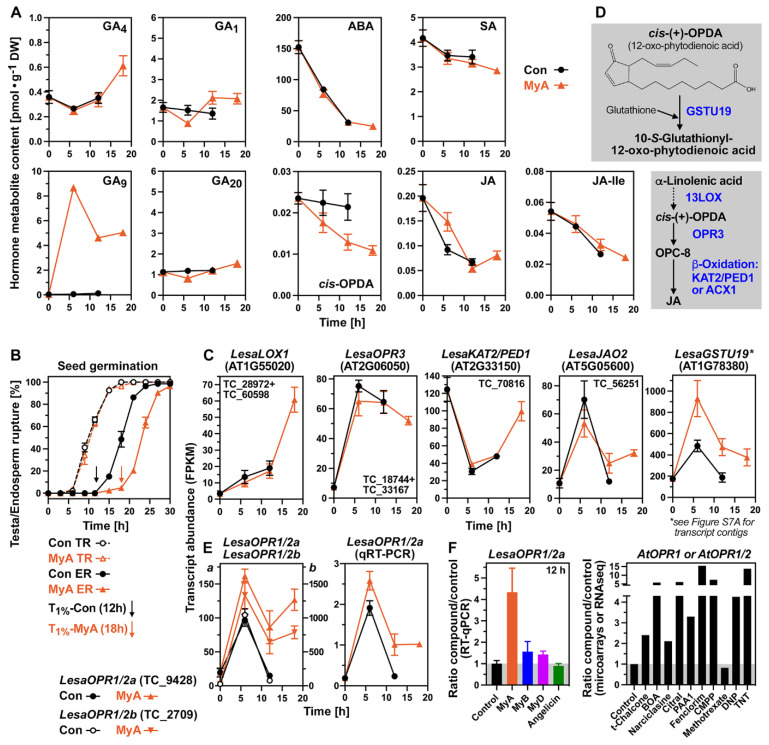
Hormone metabolites and associated gene expression in response to myrigalone A (MyA) treatment during *Lepidium sativum* seed germination. (**A**) Temporal patterns of endogenous hormone metabolites in whole seeds during germination at 20 °C without (Con) or with 0.5 mM MyA added. Bioactive gibberellins (GA_4_ and GA_1_) and their direct precursors (GA_9_ and GA_20_, respectively) are presented (see Figure S4 for other GA metabolites). Other hormones presented are *cis-S*(+)-abscisic acid (ABA), salicylic acid (SA), *cis*-(+)-12-oxophytodienoic acid (OPDA), jasmonic acid (JA) and it’s isoleucine conjugate (JA-Ile). Mean ± SEM values of five biological replicates. (**B**) MyA treatment does not affect the kinetics of testa rupture (TR) but delays the onset of endosperm rupture (ER) of the seed populations, as indicated by the arrows indicating the time when 1% ER was observed (T_1%_). (**C**) Expression patterns of *L. sativum* (*Lesa*) genes involved in OPDA and JA metabolism as affected by the MyA treatment of germinating seeds. The names of *L. sativum* (*Lesa*) genes and the corresponding *A. thaliana* orthologs (AGI in brackets) are provided; see abbreviations for full names of genes. (**D**) Simplified scheme of jasmonate biosynthesis and OPDA conjugation to glutathione by GSTU19. (**E**) *LesaOPR1/2a* and *LesaOPR1/2b* transcript expression patterns. (**F**) Effects of treatments with various compounds on *OPR1/2* transcript expression; see main text for details. Transcript abundances for *L. sativum* are presented as mean ± SEM values based on 4–5 (FPKM) and 3 (qRT-PCR) biological replicates; relevant transcript contigs (TC-IDs) are included for each graph.

Samples for the hormone and transcriptome analyses were prepared from whole *L. sativum* seeds during the time course of germination (Figure S3): dry seeds (0 h), 6 h imbibed seeds (onset of TR in the seed populations), 12 h imbibed seeds (onset of ER in the control (water) seed populations), and for MyA-treated seeds, in addition, 18 h imbibed seeds (onset of ER in the MyA-treated seed populations). This sampling scheme (Figure S3) enabled comparisons at two physically identical time points (6 h, 12 h) and, in addition, at a physiologically identical point during germination at which the seed populations are at the onset of ER (12 h for control, 18 h for MyA). To investigate how MyA affects the seed’s hormonal homeostasis, we quantified the contents of endogenous hormone metabolites during cress seed germination (Figure 2A and Figure S4). Earlier work demonstrated that upon MyA treatment, the GA precursor GA_9_ accumulates in the seed’s RAD (radicle plus lower hypocotyl) and CAP (micropylar endosperm) compartments [37,38]. Consistent with this, we found in our whole-seed analysis that GA_9_ accumulated 145-fold at 6 h and 35-fold at 12 h upon MyA treatment (Figure 2A). In addition, we show here that also GA_5_ accumulated >13-fold upon MyA treatment, but all other GA metabolite patterns did not appreciably differ between MyA and the control (Figure S4). Most importantly, MyA did not appreciably affect the whole seed’s bioactive GA_4_ and GA_1_ contents (Figure 2A). There was also no appreciable effect of MyA on the whole seed ABA and salicylic acid (SA) contents (Figure 2A). *cis*-(+)-12-oxophytodienoic acid (OPDA) is not only a precursor in the biosynthesis of jasmonic acid (JA) and its isoleucine conjugate (JA-Ile), but also an oxylipin signalling molecule on its own right [41]. OPDA remained roughly constant during cress seed germination (control) while JA and JA-Ile contents declined steadily (Figure 2A). In contrast to the control, there was a decline in OPDA over time and slightly elevated JA content at the 6 h time point upon MyA treatment (Figure 2A).

**Figure 3:**
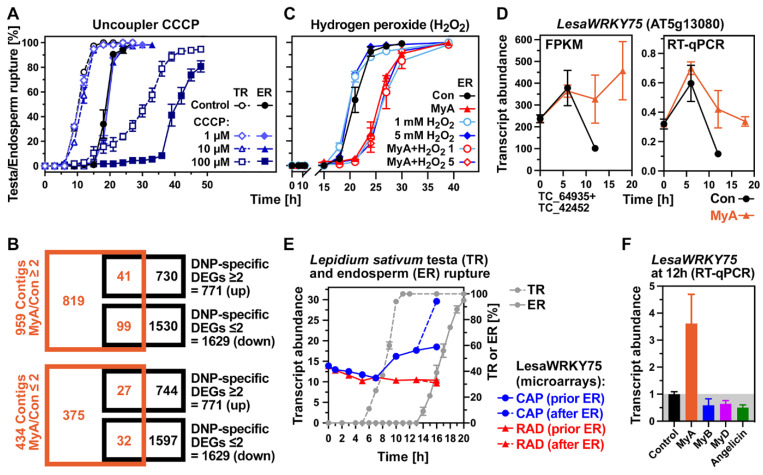
The effects of uncoupler and hydrogen peroxide (H_2_O_2_) on *Lepidium sativum* (cress) seed germination and of MyA-induced WRKY75-mediated oxidative signalling. (**A**) Kinetics of testa rupture (TR) and endosperm rupture (ER) during cress seed germination without (Control) and with the uncoupler CCPP added in the concentrations indicated. (**B**) Comparison of numbers of differentially expressed *L. sativum* transcript contigs in MyA-treated seeds with differentially expressed *A. thaliana* genes in uncoupler (DNP)-treated seeds. (**C**) Kinetics of ER during cress seed germination as affected by MyA (0.5 mM) and H_2_O_2_ added in the concentrations indicated. (**D**) Expression patterns of *WRKY75* transcripts upon treatment of cress seeds with MyA. (**E**) Spatiotemporal expression pattern of *WRKY75* during cress seed germination. (**F**) Relative *WRKY75* transcript abundance comparisons of cress seeds treated with MyA or other compounds. Transcript abundance mean ± SEM values of 4–5 (FPKM) and 3 (qRT-PCR) biological replicates are presented.

**Figure 4:**
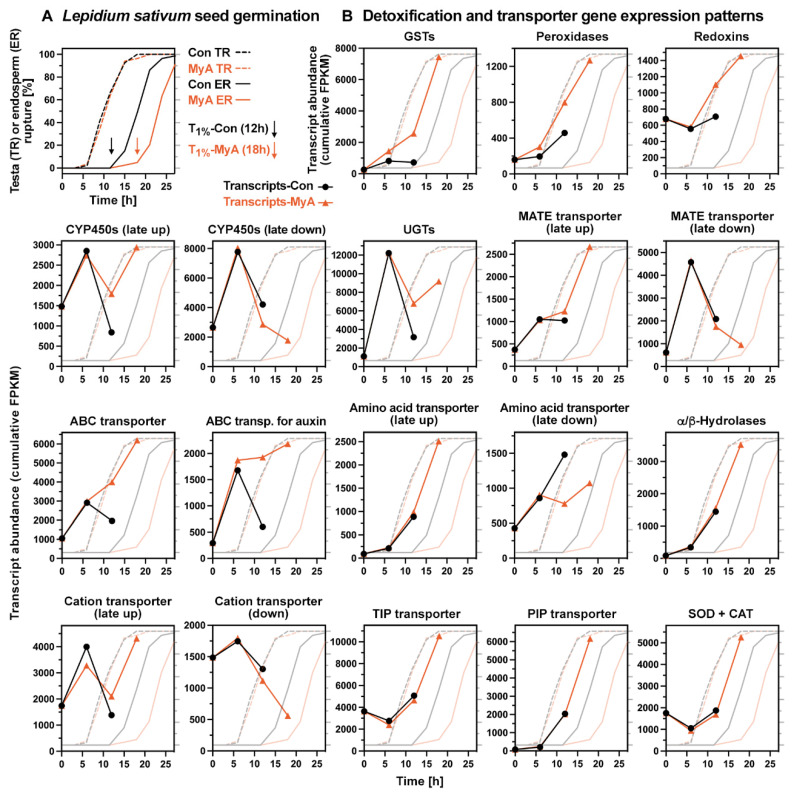
Phased induction of the seed’s detoxification programme by the phytotoxin MyA. (**A**) The kinetics of *Lepidium sativum* testa rupture (TR) and subsequent endosperm rupture (ER) without (Con) or with 0.5 mM MyA added. (**B**) Cumulative transcript expression patterns of detoxification and transporter gene groups regulated by MyA during *L. sativum* seed germination. Note that the cumulative FPKM values of differentially expressed transcript contigs (Data S2) are presented. For examples of MyA effects on specific genes (see Figure 2, Figure 3, Figure 4, Figure 5 and Figure 6 and Figures S6–S17).

**Figure 5:**
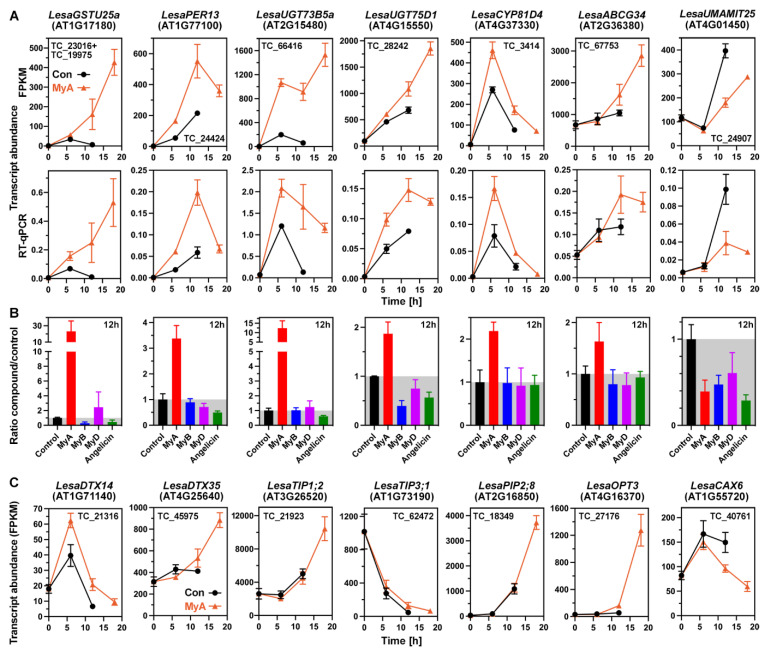
The effect of myrigalone A (MyA) and other compounds on the expression patterns of detoxification and transporter genes during *Lepidium sativum* (cress) seed germination. (**A**) Expression patterns of detoxification genes as affected by the MyA treatment of germinating seeds. (**B**) Relative transcript abundance ratios (compound/control) at 12 h during cress seed germination obtained by RT-qPCR analysis. (**C**) Expression patterns of transporter genes as affected by the MyA treatment of germinating seeds. The names of *L. sativum* (*Lesa*) genes are associated with the corresponding *A. thaliana* orthologs (AGI in brackets); see abbreviations for full names of genes. Transcript abundances are presented as mean ± SEM values (relevant transcript contigs (TC-IDs) included in each graph) based on 4–5 (FPKM) and 3 (qRT-PCR) biological replicates.

**Figure 6:**
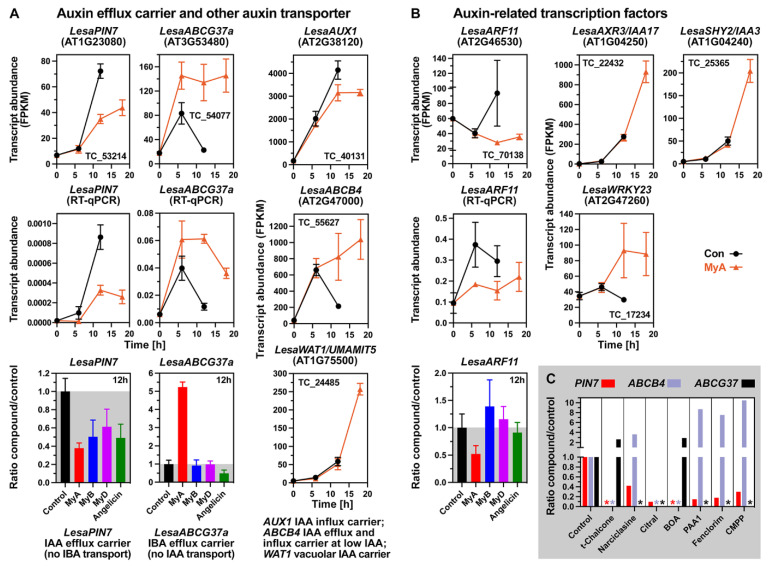
The effect of myrigalone A (MyA) and other compounds on the expression patterns of auxin-related transporter and signalling genes during *Lepidium sativum* (cress) seed germination. (**A**) Expression patterns of auxin transporter genes as affected by treatment of germinating cress seeds with MyA or other compounds. The specificity of the auxin transporters for either indole-3-acetic acid (IAA) or indole-3-butyric acid (IBA) is indicated. (**B**) Expression patterns of auxin signalling genes and the TF WRKY23. The names of *L. sativum* (*Lesa*) genes are associated with the corresponding *A. thaliana* orthologs (AGI in brackets); see abbreviations for full names of genes. Transcript abundances for *L. sativum* are presented as mean ± SEM values based on 4–5 (FPKM) and 3 (qRT-PCR) biological replicates; relevant transcript contigs (TC-IDs) are included for each graph. (**C**) Relative expression of *PIN7*, *ABCB4* and *ABCG37* upon treatment of *A. thaliana* seedlings with various compounds.

For the transcriptome (RNAseq) analysis, the same sampling scheme with RNA extraction from whole seeds was used to compare the MyA-treated and untreated (control) populations (Figure S3). Libraries of ~26 million 150 bp paired-end reads were generated for 30 samples (5 replicates for 6 samples) and 40,907 transcript assemblies each with a minimum length of 200 bp were generated as described in the methods. Their read counts were analysed as FPKM (fragments per kilobase per million; Supplementary Excel file Data S1), and the similarity of all samples was compared by PCA (Figure S5). The PCA analysis revealed that the 5th MyA replicate at 18 h (M18-5) was an outlier, and it was therefore excluded from the further analysis. The other replicates clustered together in that the principal components PC1 and PC2 accounted for 37% and 24% of the observed variance (Figure S5). Transcript contigs (assembled fragments) were selected using edgeR [42] or DESeq2 [43] based on the criterion of a log2 fold change greater than 1 (false discovery rate < 0.05) and by using all the transcripts which had minimally 5 counts per million transcripts for 4 replications. This analysis of differentially expressed transcript contigs showed that there was no large change by MyA in the transcriptomes at 6 h, as only 12 up-regulated and 4 down-regulated contigs were identified (Supplementary Excel file Data S2). Major MyA-triggered changes occurred after 6 h, and 4723 up-regulated and 27 down-regulated contigs were selected at the 12 h time point (Table 1). Comparison at the similar physiological time points between 18 h MyA-treated and 12 h control seeds identified 1341 up-regulated and 491 down-regulated contigs. Further analysis using BLAST and GO annotation (Data S1) led to a set of selected contigs, 180 up-regulated by MyA at 12 h, 24 down-regulated at 12 h, 889 up-regulated at 18 h and 419 down-regulated at 18 h, which showed similarity to either *Arabidopsis* or *Brassica* gene sequences (Table 1, Data S2). Considering the overlaps between these lists, this provided a total of 959 transcript contigs with a higher abundance (≥2-fold) upon MyA-treatment and 434 transcript contigs with a lower abundance (≤2-fold) upon MyA-treatment at the 12 h and 18 h timepoints (Data S2). These lists of MyA-regulated *L. sativum* transcripts were large enough for targeted analysis of major groups according to their biological functions. Overall, many of the up-regulated transcript contigs were annotated as stress-responsive (especially oxidative stress or pathogen defense) and xenobiotic detoxification response, while hormone-related contigs were either up- or down-regulated by MyA (Data S2). The naming of identified *L. sativum* (*Lesa*) transcripts was as described earlier [40,44] and accompanied by the transcript contig ID and by adding the AGI of the putative *A. thaliana* ortholog in brackets (Figure 2, Figure 3, Figure 4, Figure 5 and Figure 6 and Figures S6–S17).

### 2.3. MyA-Regulated OPDA Reductases, Oxylipin Pathway and Hormone Transporter Genes

The *L. sativum* OPDA reductase genes *LesaOPR1/2a*, *LesaOPR1/2b* and *LesaOPR3* were up-regulated (5 to 43-fold at 6 h) during the early phase of seed germination (Figure 2B–E). The three *A. thaliana* orthologs of these OPDA reductases *AtOPR1*, *AtOPR2* and *AtOPR3* all have enzymatic activities towards TNT and play roles in xenobiotic detoxification [45]. The *OPR1* and *OPR2* transcript sequences are very similar in both species and can not be distinguished in *A. thaliana* microarrays where a combined 14-fold up-regulation was observed in TNT-treated seedling roots [8]. In contrast to this, the *OPR3* gene was not up-regulated in seedlings by TNT [45] and was identified as the OPDA reductase implicated in JA biosynthesis [41]. Figure 2C shows that MyA treatment did not affect *LesaOPR3* expression, but up-regulated three oxylipin pathway genes at the late timepoint (18 h): 13-lipoxygenase (*LOX1*), 3-ketoacyl-CoA thiolase (*KAT2/PED1*) and JA oxidase (*JAO2*). In germinating cress seeds, these three genes are mainly expressed in the CAP (Figure S6A). KAT2/PED1 is known for its β-oxidation activity in the jasmonate pathway [41]. The observed reduction in OPDA contents upon MyA treatment (Figure 2A) could therefore involve enhanced JA production combined with subsequent JA oxidation (Figure 2C,D). Another possibility is OPDA conjugation to glutathione by glutathione-*S*-transferase GSTU19 (Figure 2D) as it was described for *A. thaliana* seedling roots [46]. In agreement with a role for OPDA glutathionylation, transcripts of *LesaGSTU19* and of *LesaGSH1*, the glutathione producing enzyme, were early up-regulated by MyA (Figure 2C and Figure S7). *LesaGSTU19* is also an example of a gene for which several transcript contigs were obtained (Figure S7A). In these cases, sequence comparisons were conducted, and if the contigs were from the same gene, as is the case for the three *LesaGSTU19* transcript contigs, they were combined (Figure S7A). The expression patterns of many genes, including *LesaGSTU19* (Figure S7A), were verified by RT-qPCR. In other cases, multiple genes are obtained as was the case for *LesaGSTU25a* and *LesaGSTU25b* (Figure S7B). Multiple genes are expected due to known gene duplications and polyploidisation events in the evolutionary history of the L. sativum genome [40,44,47]. We propose that the observed decline in the OPDA contents upon MyA treatment is mainly due to OPDA glutathionylation by MyA-induced LesaGSTU19 (Figure 2).

While MyA did not affect *LesaOPR3* expression, it enhanced *LesaOPR1/2a* and *LesaOPR1/2b* expression and prevented the decline in transcript abundance at 12 h (Figure 2E). In the late phase (12 to 18 h) >4-fold higher *LesaOPR1/2* transcript levels were observed upon MyA treatment. This was specific for MyA and not observed in germinating cress seeds treated with MyB, MyD and angelicin (Figure 2F). Table 2 summarises RT-qPCR results for selected MyA-regulated genes upon treatment with the biologically inactive dihydrochalcone MyB and chalcone MyD, and with germination-inhibiting angelicin (Figure 1 and Figure S1). Figure 2F also shows that the *OPR1/2* genes are also up-regulated in *A. thaliana* seedlings by very different allelochemicals, phytochemicals and xenobiotics. This comparison was also conducted for other MyA-regulated genes (Table 3 and Table 4) and was achieved by data mining of published transcriptomes for the responses to treatments with *trans*-chalcone [19], benzoxazolin-2(*3H*)-one (BOA) [4], narciclasine (NCS) [20], citral [18], A_1_-phytoprostane (PPA_1_) [48], the herbicide safeners fenclorim and CMPP [13], methotrexate (MTX) and 2,4-dinitrophenol (DNP) [31], and TNT [8]. While *OPR3* was not induced by MyA or any of these other compounds, *OPR1/2* was induced by MyA and all these compounds apart from MyB, MyD, angelicin and MXT (Figure 2F). Up-regulation of the *OPR1/2* genes, therefore, seems part of a more general detoxification response towards phytotoxins including MyA.

During the late phase of cress seed germination, MyA regulated GA, ABA and 1-aminocyclopropane-1-carboxylic acid (ACC) transporter genes (Figure S6B). Examples of these from the GA transporting nitrate/peptide transporter family (NPF) proteins [49] include *LesaNPF6.2* and the CAP-expressed *LesaNPF6.3* for which the up-regulation was further enhanced by MyA (Figure S6B). The transcript abundance of the ABA influx transporter ABCG40, which transports endosperm-produced ABA into the embryo [50], was also up-regulated by MyA, while the expression of the ABA efflux transporter ABCG25 was not affected (Figure S6B). For the ACC, tyrosine and asparagine transporting lysine histidine transporter LHT1 [51], higher *LesaLHT1* transcript levels were observed in germinating cress seeds in the CAP as compared to the RAD (Figure S6B). MyA, MyB, MyD and angelicin all reduced *LesaLHT1* expression in germinating cress seeds at 12 h (Figure S6B). The effects of the other compounds (Figure S2) on the expression of these transporters in *A. thaliana* seedlings differed in that the GA transporters NPF6.2 and NPF6.3 were either not regulated or down-regulated by these compounds (Table 3). As for MyA, *ABCG40* was up-regulated by almost all of these compounds, while *LHT1* was down-regulated by some compounds (Table 3). Expression of the OPDA transporter *COMATOSE* (*CTS*) gene was not affected by MyA or any of the other compounds except for citral (Table 3). It therefore seems from these examples already, that specific interference with tissue-specific hormone transport is part of MyA’s action.

### 2.4. Evaluation of the Roles of Proposed Myrigalone Bioactivities during Seed Germination

Natural chalcones and derivatives have numerous bioactivities and molecular targets [19,32,33]. Some chalcone derivatives inhibit p-hydroxyphenylpyruvate dioxygenase (HPPD) enzyme activity. This is not the case for MyA [11] and MyA also does not cause seedling bleaching as does the HPPD-targeting herbicide sulcotrione [37]. Recent work with *A. thaliana* seedlings showed that *trans*-chalcone also did not inhibit HPPD activity, it did, however, cause seedling bleaching and was proposed to be a protoxin that is converted to a HPPD inhibitor *in planta* [19]. These authors also showed that *AtHPPD* transcripts were early up-regulated in seedling roots and shoots upon treatment with *trans*-chalcone. In contrast to this, *LesaHPPD* transcript levels steadily declined during *L. sativum* seed germination and were not affected by MyA treatment (Figure S8A). HPPD transcript levels also steadily declined during *A. thaliana* seed germination, and interestingly treatment with the uncoupler DNP caused their 7.3-fold up-regulation in seeds [31] (Figure S8A). In contrast to *trans*-chalcone and DNP, none of the other compounds investigated caused HPPD up-regulation (≥2-fold) in *A. thaliana* seedlings (Table 4). In summary, we conclude that HPPD is not a MyA target.

DNP and carbonyl cyanide *m*-chlorophenyl hydrazone (CCCP) are both classical uncouplers of oxidative phosphorylation [31,52]. Uncoupler activity has been demonstrated for MyA and MyB in a rat liver mitochondria assay system [34]. To investigate if uncoupling activity plays a role in the MyA-mediated inhibition of *L. sativum* seed germination, we compared the effects of MyA with the uncoupler CCCP. While up to 1 mM MyA delayed ER without affecting TR, MyB affected neither of these rupture events, while 100 µM CCCP delayed both TR and ER (Figure 1 and Figure 3A). We then compared which DEGs identified in the transcriptomes overlap between imbibed seeds treated with the uncoupler DNP [31] and with MyA (Figure 3B). Of the DNP-specific up-regulated DEGs (771), only 5.3% (41) were also up-regulated in MyA-treated seeds. Among these was none related to the oxidative pentose phosphate pathway, glycolysis, tricarboxylic acid cycle or fatty acid biosynthesis known to be up-regulated during CCCP-mediated uncoupling [52]. Similarly, of the DNP-specific down-regulated DEGs (1629), only 2.0% (32) were also down-regulated in MyA-treated seeds (Figure 3B). We therefore conclude that uncoupling of oxidative phosphorylation is not a major mechanism by which MyA inhibits seed germination.

Earlier work with mammalian cell lines also showed that myrigalones and other dihydrochalcones are antioxidants with radical scavenging properties [35,53]. Reactive oxygen species (ROS) signalling is known to regulate seed germination in many species [54,55] and our previous work demonstrated that MyA acts as a scavenger of apoplastic ROS in imbibed *L. sativum* seeds [37]. To further assess how MyA acts as a ROS scavenger during cress seed germination, the effect of hydrogen peroxide (H_2_O_2_) was investigated. Dose-response assays showed that 1–100 mM H_2_O_2_ promoted TR and ER to varying degrees and that 1–5 mM H_2_O_2_ promoted ER at a similar level as 10–100 µM GA (Figure S8B). As shown in Figure 3C, 1 and 5 mM H_2_O_2_ alone slightly promoted ER, but the delay in ER by MyA was not rescued by simultaneous treatment with MyA plus H_2_O_2_. This tendency was also confirmed with 50 mM H_2_O_2_ (Figure S8B). The importance of oxidative stress, ROS scavenging and H_2_O_2_ signalling were further evident from the ROS scavenging ascorbate and glutathione antioxidant systems [55,56,57]. These systems were up-regulated by MyA during the late germination phase between 12 h and 18 h (Figure S9). Among others, MyA up-regulated ascorbate peroxidases (APX), dehydroascorbate reductase (DHAR) and monodehydroascorbate reductase (MDAR), glutaredoxins, superoxide dismutases (SOD), and catalase (CAT). SOD genes (*FDS1*) were also up-regulated in seeds treated with angelicin (Figure S9B, Table 2), but not by any of the other compounds in *A. thaliana* seedlings (Table 4). *DHAR2* was also up-regulated in seedlings by most of the other chemical compounds, but many other MyA-induced genes of the ascorbate and glutathione antioxidant systems were not regulated by other compounds (Table 4). As H_2_O_2_ has been shown to stimulate GA biosynthesis [58] and the effects of GA or H_2_O_2_ were evident as promotion of TR (Figure 1B and Figure S8B), the MyA-mediated inhibition of ER may be controlled downstream of GA and H_2_O_2_ signalling.

Candidates for this are stress-responsive transcription factors (TFs) which are known to be involved in H_2_O_2_ and GA signalling [56]. Among TFs up-regulated by MyA in germinating cress seeds at the 12 h time point were WRKY75, WRKY23 and WRKY6 (Figure 3 and Figure S10A,E). Their transcript abundances were higher at the 12 h and 18 h timepoints upon MyA treatment, and they were mainly expressed in the CAP. WRKY75 expression is known to be induced by GA, H_2_O_2_ and SA, and repressed by JA [59,60]. WRKY75 has been shown to be involved in ROS and GA signalling and to physically interact with DELLA proteins. SIB1 and SIB2 also physically interact with WRKY75 to inhibit its activity in seed germination [61]. While *LesaWRKY75* expression was up-regulated by MyA in germinating seeds, neither MyB, MyD, nor angelicin stimulated its expression (Figure 3F). The transcript abundances of *LesaSIB1* and CAP-expressed *LesaSIB2* were lower upon MyA treatment (Figure S10F), suggesting that MyA responses involve WRKY75 activity in the CAP. Several bHLH, ERF and NAC TFs were up-regulated by MyA at the 12 h and 18 h time points (Figure S10) [54,56,62]. Among them are bHLH38, important for regulating iron homeostasis, bHLH129 with CAP-specific expression, ERF2, ERF20, and several NAC TFs (Figure S10, Data S2): The membrane-associated NAC005, the metabolism regulator NAC032, the development regulator NAC081, and the xenobiotic detoxification and low-oxygen responsive regulator NAC102 [56,62,63]. In contrast to these TFs, the mainly CAP-expressed ABA-associated ABI3 and ABI5 TFs were down-regulated during the late germination phase and this down-regulation was further intensified by MyA (Figure S10F). Among the NAC and ERF TFs, which were up-regulated by MyA in *L. sativum* germinating seeds, NAC102 was the one also up-regulated by most of the chemical compounds investigated in *A. thaliana* seedlings (Table 4). In addition, most of the allelochemicals also up-regulated NAC081 and ERF20 in seedlings (Table 4). These TFs, therefore, may play more general roles in inducing the detoxification programme in response to phytochemicals. WRKY23 is a component of the transcriptional network which controls auxin distribution patterns [64,65] together with auxin signalling TFs (Table 3). Their regulation by MyA is discussed later in the context of auxin signalling and transport as a potential target of MyA action.

### 2.5. Phased Induction of the Seed’s Detoxification Programme by the Phytotoxin MyA

The transcriptome analysis of *L. sativum* seed germination revealed that the natural phytotoxin MyA triggered the phased induction of a typical detoxification programme (Figure 4). MyA induced the early up-regulation of glutathione-producing glutamate-cysteine ligase (*GSH1*), glutathione-*S*-transferases of the “Tau” class (GSTU), and peroxidases gene expression at the 6 h and 12 h time points (Figure 5A, Figures S7 and S9). GSH is required for the glutathione cycle (Figure S9) and for GST-catalysed glutathionylation reactions leading to *S*-conjugates with pesticides, allelochemicals or endogenously produced metabolites. These conjugates are usually further metabolised or transported into vacuoles for detoxification and storage [2,9,46]. This includes possible OPDA glutathionylation by MyA-induced *LesaGSTU19* in germinating cress seeds (Figure 2B). *LesaGSTU25*, *LesaGSTU22*, *LesaGSTU1*, and *LesaGSTU8* are examples of genes that were up-regulated by MyA treatment already at the 6 h time point (Figure 5A and Figure S7B). GSTU25 is known to be involved in the detoxification of the explosive TNT by catalysing the formation of glutathione-TNT conjugates [66]. Expression of class III peroxidases *LesaPER13* (Figure 5A) and *LesaPER45* (Figure S7C) was also up-regulated by MyA in the early (6 h) and late (18 h) phase of germination, respectively. Class III peroxidases are known to serve specific roles in development and stress responses, including reproduction (PER13), TNT stress (PER45), and testa and endosperm rupture during *A. thaliana* and *L. sativum* seed germination [67,68]. Up-regulation of redoxins, including glutaredoxin, by MyA treatment, preceded the up-regulation of SOD, CAT, and the ascorbate pathway enzymes (Figure 4 and Figure S9). The enhancing effect of MyA on the up-regulation of *LesaPER13*, *LesaGSTU25* and *LesaGSTU19* was unique for MyA and not observed in cress seeds treated with MyB, MyD, angelicin, while *LesaPER45* was also induced by angelicin (Figure 5 and Figure S7, Table 2). The transcript abundance of the plantacyanin *LesaPCY* were CAP-specific up-regulated by MyA during all phases of germination (Figure S7C). Plantacyanins are blue copper proteins and PCY has recently been demonstrated to be a key regulator of *A. thaliana* seed germination linking environmental factors and hormones [69].

UDP-glycosyltransferases (UGTs) catalyse the transfer of UDP-activated sugars to acceptor molecules, the aglycones include xenobiotics, secondary metabolites and plant hormones [8,9,70]. *LesaUGT73B5* and *LesaUGT75D1* were specifically expressed in the CAP and early up-regulated by MyA in germinating *L. sativum* seeds (Figure 5A and Figure S11). *LesaUGT73B5* and *LesaUGT75D1* were not up-regulated by MyB, MyD, and angelicin (Figure 5B). UGT73B5 is known to be up-regulated by ROS and SA, and is part of the detoxification mechanism of TNT and other compounds [8,71]. Overexpression of UGT75D1 has been shown to increase abiotic stress tolerance of seed germination [70]. These authors proposed that UGT75D1 has the auxin indole-3-butyric acid (IBA) as a preferred substrate and that it is involved in auxin-ABA crosstalk in seeds. *LesaUGT74E2* and *LesaUGT75B1* are mainly expressed in the CAP and, together with other UGTs, were up-regulated by MyA during the late phase of germination (Figure S11). UGT74E2 and UGT75B1 are known to be involved in the control of auxin homeostasis, auxin signalling and transport in the root, and also have been proposed to have IBA as their preferred substrate [72]. UGT74E2 was not up-regulated by MyB, MyD and angelicin at 12 h or 18 h (Table 2) and was down-regulated by MyB and MyD at 6 h (Figure S11B).

Cytochrome P450 monooxygenases (CYP450) typically conduct hydroxylation and oxygenation reactions in secondary metabolism or detoxification pathways [9]. *LesaCYP81D4* was early up-regulated by MyA (Figure 5A), while *LesaCYP81D8*, *LesaCYP71B26*, *LesaCYP75A32* and *LesaCYP76C2* were late up-regulated by MyA in germinating cress seeds (Figure S12A). In contrast to MyA, *LesaCYP81D4* and *LesaCYP81D8* were not up-regulated in seeds by MyB, MyD, and angelicin (Figure 5B, Table 2). CYP81D and CYP71B are among the five identified contigs in blackgrass proposed to be involved in non-target-site herbicide resistance [73]. CYP76C2 is known to be implicated in the metabolism of monoterpenes and phenylurea herbicides [74] and is in *A. thaliana* seedlings up-regulated by treatment with citral [18]. Several CYP450s, including *LesaCYP78A7* and *LesaCYP75B1/TT7* were down-regulated by MyA in germinating cress seeds (Figure S12A). NADPH:cytochrome P450 reductases, encoded by the genes *ATR1* and *ATR2* in *A. thaliana*, enable electron transfer from NADPH to cytochrome P450 [75]. The transcript levels of *LesaATR1* declined in germinating cress seeds, but in the late phase, remained at a higher level upon MyA treatment (Figure S12B). Several other GSTs, UGTs, peroxidases, and CYP450s contributed to the general up-regulation pattern by MyA (Figure 4 and Figures S9–S12, Data S2). In addition, other enzymes such α/β-hydrolases are known to be part of the plant’s detoxification programme [9] and were up-regulated by MyA (Figure 4).

### 2.6. MyA Interferes with Transporter Gene Expression in Germinating Cress Seeds

Multidrug and toxic compound extrusion (MATE) transporters in plants, also known as DETOXIFICATION (DTX) proteins, are integral membrane proteins involved in an array of functions, including secondary metabolite transport and xenobiotic detoxification [76]. Two MyA-induced expression patterns, either late up-regulated or late down-regulated, were identified for MATE transporters in germinating *L. sativum* seeds (Figure 4). *LesaDTX14* was early up-regulated mainly in the CAP of germinating seeds and higher expressed upon MyA-treatment (Figure 5C and Figure S13A). DTX14 is known as a xenobiotic extrusion transporter [77]. The CAP-specific expressed *LesaDTX35/FFT* was up-regulated by MyA in germinating cress seeds (Figure 5C and Figure S13A). DTX35 is known to function as a tonoplast chloride/anion channel [78]. It has been proposed to be a flavonoid transporter involved in seed development and germination [79]. Other MATE transporters affected by MyA in germinating cress seeds include the late up-regulated *LesaDTX9*, *LesaDTX16*, and *LesaDTX2*, and the late down-regulated *LesaDTX4*, *LesaDTX51*, and *LesaDTX45* (Figure S13A). DTX45 is known to antagonise local ABA signalling and distribution in *A. thaliana* seedlings [80].

ATP-binding cassette (ABC) transporters use the energy from ATP hydrolysis to drive the transport of diverse substrates [81]. The transcript abundances of all differentially expressed ABC transporters in germinating *L. sativum* seeds were up-regulated by MyA (Figure 4). This includes several auxin-transporting ABC transporters, which are discussed in the next section. The up-regulation of *LesaABCG34* expression in the CAP during cress germination was further enhanced by MyA (Figure 5A and Figure S13B). ABCG34 is known as a transporter of monolignols and secretion of defence compounds by roots [50,81]. *LesaABCG34* up-regulation in MyA-treated cress seeds was unique for MyA and not observed for MyB, MyD and angelicin (Figure 5B). In *A. thaliana* seedlings, *AtABCG34* was also up-regulated in shoots upon *trans*-chalcone treatment [19], but not by any of the other compounds investigated (Table 4). Transcripts of *LesaABCG40* (ABA influx carrier, Figure S6), and *LesaABCG14* (Figure S13B) were up-regulated in germinating cress seeds by MyA. ABCG14 is known to control the root-to-shoot translocation of cytokinins [50,82,83]. In *A. thaliana* seedlings, *AtABCG14* was not up-regulated by any of the compounds investigated (Table 4). *LesaABCG14/MDR12*, *LesaABCG7*, and *LesaABCG12* were higher expressed upon MyA treatment during the late phase of germination (Figure S13B). ABCG12 is known as a transporter of cutin and cuticular wax monomers and ABCB14/MDR12 as a malate transporter [81]. ABCB14/MDR12 was also suggested to function as a facultative auxin transporter but lacks the required conserved D/E-P motif [84].

Amino acid transporters are the main mediators of nitrogen distribution into developing seeds and between tissues during germination [85,86,87]. In agreement with the importance of amino acid transport in germinating *L. sativum* seeds, transcripts of several UMAMIT-type amino acid transporter genes were regulated by MyA (Figure S14A). While transcript levels of *LesaUMAMIT30* and several other *LesaUMAMIT* genes, *LesaGDU7*, and the γ-aminobutyric acid (GABA) transporter *LesaGAT1* were elevated upon MyA treatment during late seed germination, those of *LesaUMAMIT25* and *LesaUMAMIT12* were reduced (Figure 5A and Figure S14A). Similar to MyA, *LesaUMAMIT25* was also down-regulated by MyB, MyD and angelicin (Figure 5B). Among the ion transporters up-regulated by MyA in germinating *L. sativum* seeds were the mainly CAP-expressed oligopeptide transporter *LesaOPT3* (Figure 5B and Figure S14B), several heavy metal-associated isoprenylated plant proteins including *LesaHIPP6*, and phosphate transporters including *LesaPHT1;4* (Figure S14B). OPT3 is known as an important component of the seedling iron-signalling network, and plays a critical role in seed iron transport, homeostasis and nutrition [88]. HIPPs are involved in heavy metal homeostasis, detoxification mechanisms and stress responses [88]. In contrast to these up-regulated cation transporters, the expression of many other cation transporters was down-regulated by MyA during the late phase of cress seed germination (Figure 4). Among them were the cation/proton exchanger *LesaCAX6* (Figure 5C), the RAD-expressed vacuolar iron transporter *LesaVIT*, and the CAP-expressed potassium uptake permease *LesaKUP9* (Figure S14B), and several others (Data S2). Several of these cation transporters are known to be involved in ROS signalling and abiotic stress responses, as well as ion, osmotic and root auxin homeostasis [78,88,89].

Transcripts of aquaporins, tonoplast intrinsic proteins (TIP) and plasma membrane intrinsic proteins (PIP) were up-regulated (*LesaTIP1;2*, *LesaPIP2;8*, *LesaPIP2;6*, *LesaPIP2;2*, *LesaPIP1;3*) or down-regulated (*LesaTIP3;1*) in germinating cress seeds (Figure 5C and Figure S14C). MyA-treatment enhanced the up-regulation for all these PIPs during the late phase of germination (Figure 4) and for the very abundant *LesaTIP1;2* (Figure 5C), while it retarded the down-regulation of *LesaTIP3;1* (Figure 5C). Aquaporins assist with water relations which are especially important during seed germination and in responses to abiotic stresses [90,91]. TIPs and PIPs transport water across membranes and may also transport other substances. An example for this is AtTIP1;2 which also transports H_2_O_2_ and is therefore involved in ROS homeostasis. The MyA-enhanced up-regulation of *LesaTIP1;2* in RAD and CAP (Figure 5C and Figure S14C) supports the conclusion that redox and ROS signalling are important for the MyA response. The down-regulation of *LesaTIP1;3* (Figure 5C) in the non-dormant *L. sativum* seeds is in agreement with the finding that ABA contents (Figure 2A) and *LesaABI3* expression (Figure S10F) declined during seed germination. In *A. thaliana* seeds, ABA signalling is known to activate *AtTIP1;3* expression via ABI3 TF activity and this contributes to seed dormancy [92]. The accumulation of aquaporins in their function as water channels supports our earlier finding that MyA enhances water uptake into cress seeds [38]. In this publication, we proposed that MyA is a soil seed bank-destroying allelochemical that secures the persistence of M. gale in its flood-prone environment. The transcriptome results are in support of this hypothesis and that MyA targets several aspects of seed germination and subsequent seedling growth. The genes which were regulated by MyA during the late germination phase are important for early seedling growth.

### 2.7. MyA Interferes with the Expression of Auxin Transport and Signalling in Germinating Seeds

Auxin biosynthesis, signalling and transport are tightly regulated to control plant development and environmental responses. This is also the case for the control of seed germination during which indole-3-acetic acid (IAA) biosynthesis is up-regulated in the embryo’s radicle, as has been shown for *L. sativum* [40,93] and *A. thaliana* [94,95]. Precise auxin distribution patterns and polar transport are achieved by members of different auxin transporter families, including for auxin transport between cells, PIN efflux carriers, AUX1/LAX influx carriers, different classes of ABC transporters, and intracellular auxin transport, PILS auxin carrier and the WAT1/UMAMIT5 protein [81,83,96,97,98]. Figure 6A shows that MyA treatment of germinating *L. sativum* seeds reduced the accumulation of *LesaPIN7* transcripts. In contrast to this, the expression of *LesaAUX1*, *LesaPIN1a*, *LesaPIN1b* and *LesaPIN2*, were only weakly reduced by MyA, and other *PIN* genes were not affected (Figure 6A and Figure S15A). PINs function as IAA efflux carriers with a coordinated cell-specific asymmetric (polar) subcellular localisation [96].

**Figure 7:**
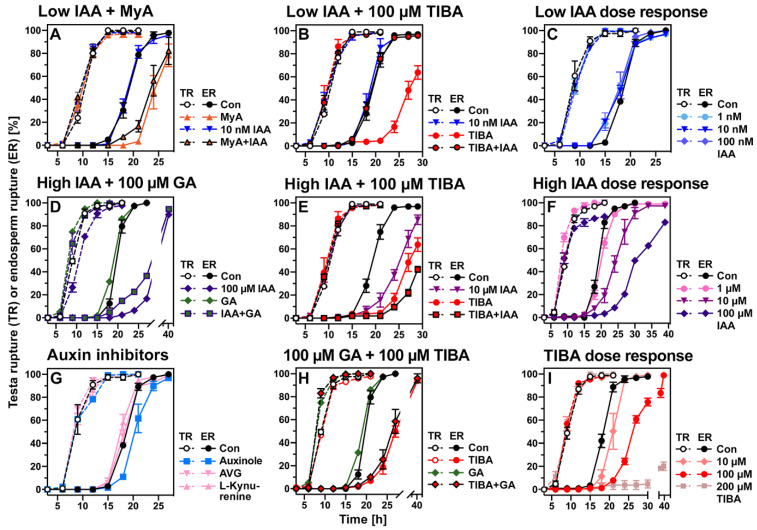
The effects of indole-3-actic acid (IAA), the auxin transport inhibitor TIBA, and other auxin-related inhibitors on *Lepidium sativum* seed germination and its inhibition by myrigalone A (MyA). (**A**) The effect of adding a low IAA concentration on the kinetics of testa rupture (TR) and subsequent endosperm rupture (ER) without (Con, control) or with 0.5 mM MyA added. Note that combined treatment with a low IAA concentration partly reverts the MyA-mediated delay of ER. (**B**) The effect of adding a low IAA concentration on the kinetics of TR and ER without (Con) or with 100 µM TIBA (2,3,5-triiodobenzoic acid) added. Note that combined treatment with a low IAA concentration fully reverts the TIBA-mediated delay of ER. (**C**) IAA dose-response of cress seed germination for the low concentration range. (**D**) The effects of IAA and gibberellin (100 µM GA_4+7_) on germination. (**E**) The effect of adding a high IAA concentration on the kinetics of TR and ER without (Con) or with 100 µM TIBA added. Note that combined treatment with a high IAA and TIBA resulted in additive ER inhibition. (**F**) IAA dose-response of cress seed germination for the high concentration range. (**G**) The effect of the TIR1 auxin receptor antagonist auxinole (100 µM) and of the auxin biosynthesis inhibitors (100 µM) aminoethoxy-vinylglycine (AVG) and L-kynurenine on cress seed germination. (**H**) The effects of TIBA and gibberellin (100 µM GA_4+7_) on germination. (**I**) TIBA dose-response of cress seed germination. Note that TIBA inhibits ER in a dose-dependent manner without affecting TR. Seeds were incubated at 20 °C in continuous white light, TR and ER scored over time, mean values ± SEM for 3 replicates each with ca. 30 seeds are shown.

To test if MyA affects PIN-mediated IAA transport, we treated germinating cress seeds with the IAA transport inhibitor 2,3,5-triiodobenzoic acid (TIBA) [99]. TIBA inhibited endosperm rupture (ER) in a dose-dependent manner without appreciably affecting testa rupture (TR), similarly as was observed for MyA (Figure 7A,I). This delay in ER timing caused by TIBA was not restored by the exogenous application of GA (Figure 7H). Treatment of germinating seeds with IAA showed a dose-dependent response: Low IAA concentrations (1–100 nM) slightly promoted ER (Figure 7C) while high IAA concentrations (1–100 µM) delayed ER (Figure 7F). The delay in ER by a high (100 µM) IAA concentration was partly rescued by simultaneous GA treatment (Figure 7D). When TIBA was combined with an inhibitory high (10 µM) IAA concentration, their combined inhibitory effect was additive, and ER was more severely delayed than that with either TIBA or IAA alone (Figure 7E). Interestingly, when TIBA was combined with a low (10 nM) IAA concentration, the kinetics of ER was the same as the control (Figure 7B). Application of a low IAA concentration therefore fully reverted the inhibitory effect of TIBA on cress seed germination. Application of IAA in a low concentration did however not revert the inhibitory effect of MyA on cress seed germination, but a slight promotion was observed (Figure 7A). Taken together, this suggested that the delay in ER by MyA was in part caused by local IAA deficiency and perturbed IAA distribution in the RAD and/or CAP tissues of germinating seeds.

Further to this, treatment of germinating *L. sativum* seeds with the TIR1 IAA-receptor antagonist auxinole [100] also caused a delay in ER without appreciably affecting TR (Figure 7G). In contrast to this, treatment with inhibitors of auxin biosynthesis had no effect (Figure 7G). Different combinations of TIR1 and AUX/IAA proteins are known to form co-receptor complexes with a wide range of auxin-binding activities. AUX/IAA repressor accumulation is known to down-regulate *ABI3* transcription and this auxin-ABA signalling promotes seed germination [95,101,102]. Transcript levels of *LesaABI3* and *LesaABI5* declined during the late phase of cress seed germination and this decline is further enhanced by MyA (Figure S10F). In agreement with this and the role of auxin signalling via AUX/IAA proteins in the MyA-mediated inhibition of cress ER, transcript accumulation of *LesaAXR3/IAA17* and *LesaSHY2/IAA3* was enhanced by MyA treatment (Figure 6B). The TF WRKY23 is part of a complex of auxin signalling AUX/IAA repressor proteins and ARF TFs, which is known to control PIN polarity and auxin distribution patterns [64,65,95,96]. It is known that AXR3/IAA17 is a component of this WRKY23-mediated auxin feedback on PIN polarity [64], and that the control root meristem growth via a regulatory circuit converges at SHY2/IAA3 to regulate PIN7 expression [103]. In agreement with a role of this regulatory complex in reducing *LesaPIN7* expression, MyA-treatment of germinating cress seeds enhanced the up-regulation of *LesaWRKY23*, *LesaAXR3/IAA17* and *LesaSHY2/IAA3* (Figure 6B), and inhibited the expression of *LesaARF11* (Figure 6B), *LesaARF1* and *LesaARF18* (Figure S16). The expression of auxin-responsive SAUR genes and the nucleoside diphosphate kinase NDPK2, known for its involvement in auxin-mediated responses, were enhanced by MyA (Figure S16). The reduction in *LesaARF11* expression by MyA in germinating cress seeds was not observed with MyB, MyD or angelicin (Figure 6B). In contrast to this, the reduction in *LesaPIN7* expression by MyA was also observed for MyB, MyD and angelicin (Figure 6A). We propose that MyA-altered auxin signalling via TIR1-AUX/IAA and WRKY23 interaction are involved in the localised perturbation of the IAA distribution in *L. sativum* seeds and that this has the expression and/or polarity of auxin transporters as a target. A major target of MyA is the IAA transporter LesaPIN7, but MyA also affects the expression of other auxin transporters.

In addition to the PIN efflux carriers, IAA transport and tissue-specific distribution are mediated by facultative IAA importers/exporters for which the transport directionality depends on the IAA concentration, examples of this are ABC transporter of the ABCB class [81,83,84]. While MyA inhibited *LesaPIN7* transcript accumulation in germinating cress seeds, it enhanced the up-regulation of mainly CAP-expressed *LesaABCB4* (Figure 6A and Figure S15D) and *LesaABCB11* transcripts (Figure S15C). ABCB4 is an IAA efflux transporter in *A. thaliana* seedling roots with reported IAA uptake activity at low IAA concentrations [104,105,106]. ABCB4 is known to stably associate with the plasma membrane and exhibits intracellular trafficking distinct from that of PIN proteins. The low (nanomolar) IAA concentrations required to induce efflux activity of ABCB4 suggest that the protein functions primarily as an efflux transporter in the root apex. MyA treatment also enhanced the expression of *LesaWAT1/UMAMIT5* in the RAD late during seed germination (Figure 6A and Figure S15D). WAT1 is a tonoplast-localised protein that functions as a vacuolar IAA transport facilitator required for auxin homeostasis [98]. PIN, ABCB4 and WAT1 are IAA-specific and do not transport IBA, but in contrast to these, ABCG37 is an IBA-specific transporter and does not transport IAA [83,107,108]. ABCG37 acts as a plasma membrane located IBA exporter and this IBA transport is not blocked by TIBA. *LesaABCG37a* and *LesaABCG37b* transcripts accumulated upon MyA treatment during the early and late phase of cress seed germination (Figure 6A and Figure S15C). In contrast to MyA which enhanced *LesaABCG37* expression in germinating cress seeds, it was not induced by MyB, MyD or angelicin (Figure 6A). Taken together, it seems likely from the transcriptome responses that interference with auxin signalling and transport are major targets of MyA for inhibiting ER during seed germination.

### 2.8. Conserved and Chemical-Specific Detoxification Response and Interference with Auxin Transport

We conclude from the transcriptome analysis that a typical detoxification programme [2,9,10] was triggered by MyA in germinating *L. sativum* seeds. This type of response requires compound sensing and signalling, which is typically followed by the phased induction of detoxification enzymes and transporters (Figure 4 and Figure 5), and in the case of MyA, also by interference with auxin transport and signalling (Figure 6 and Figure 7). On the one hand, MyA induced similar groups of detoxification genes when compared with other phytotoxic compounds, including various allelochemicals and xenobiotics (Table 2, Table 3 and Table 4). On the other hand, there are striking differences within each group in which specific genes were induced by the different compounds (Figure 1, Figures S1 and S2). The comparative transcriptome analysis conducted here (Table 2, Table 3 and Table 4) includes imbibed seeds treated with the germination-inhibiting phytotoxins MyA, angelicin [this work], MTX and DNP [31], *A. thaliana* seedlings or seedling roots treated with the allelochemicals *trans*-chalcone [19], NCS [20], citral [18], and BOA [4], as well as with PPA_1_ [48] or (root cultures) the herbicide safeners fenclorim and CMPP [13]. Among the detoxification genes up-regulated by MyA, the enzymes GSTU25, GSTU22, GSTU1, CYP81D8, UGT75B1, UGT73B2 and UGT73B5 constitute a more general response as they were up-regulated by most of the allelochemicals and xenobiotics (Table 4). None of these genes were, however, up-regulated by all compounds; GSTU25 was, for example, not up-regulated by citral (Table 4) and angelicin (Figure 5A), and GSTU22 and GSTU1 were down-regulated in citral-treated roots (Table 4). GSTU19, CYP710A1 and UGT74E2 are examples of detoxification enzymes up-regulated by some of the compounds only (Table 2 and Table 4). CYP81D4 and PER13 are examples of MyA-specific up-regulation as they were not affected by any of the other compounds (Figure 5, Table 4). PER45 expression was up-regulated in seeds by MyA and angelicin (Figure S7, Table 2), but down-regulated in seedlings and seeds by almost all other compounds (Table 4).

Further examples demonstrating diversity in responses include transporters and redox homeostasis: With the exception of DHAR2, which was up-regulated by most compounds, the antioxidant system components were up-regulated by MyA but not regulated or even down-regulated by other phytotoxins (Table 4). This pattern also became evident for many UMAMIT and aquaporin transporter genes which were up-regulated by MyA, but down-regulated or not regulated by most of the other phytotoxins (Table 4). The MATE transporter DTX14 was up-regulated by several of the compounds, while other MATE transporters revealed mixed response patterns (Table 4). In contrast to the other phytotoxins, the response of roots to citral treatment was down-regulation for most of the selected enzyme and transporter groups [109]. Citral, therefore, acts by inhibiting gene transcription and has multi-molecular target sites. Altering the water status appears to be a common target of MyA [38] and citral [109], but the interference seems to be achieved differently. MyA enhanced the up-regulation of aquaporins in cress seeds while citral down-regulated aquaporin expression (Table 4). The general conclusion from these examples is that there is a mixture of more general and more specific response patterns for the different phytotoxins.

When the entire list of 959 MyA up-regulated transcript contigs in germinating cress seeds (Data S2) was compared with the lists of genes up-regulated in seedlings by the four allelochemicals *trans*-chalcone [19], NCS [20], citral [18], and BOA [4], a core set of 12 common allelochemical responsive genes were identified. These were *GSTU25*, *GSTU1*, *CYP81D8*, *UGT75B1*, *UGT73B2*, *UGT73B5*, *OPR1/2*, α/β-hydrolase (AT4G24160), *PMAT1*, *SAT1*, *tolB* and *NAC102* (Table 4, Figure S17). Of these, PMAT1 is a malonyltransferase important in phenolic-xenobiotic metabolism [110], SAT1 is a component of OPDA-related redox homeostasis and oxylipin signalling [111], the α/β-hydrolase (AT4G24160) plays a role in maintaining lipid homeostasis [112], and tolB is also up-regulated in TNT-treated seedlings [8]. In contrast to α/β-hydrolase (AT4G24160), which was up-regulated by all phytotoxins, up-regulation of the α/β-hydrolase BDG1 was MyA-specific (Figure S17B, Table 4). BDG1 is involved in cutin production required for the endosperm-associated cuticle in seeds [113]. OPR1/2 (Figure 2) and several other oxidoreductases were also up-regulated by MyA and most of the allelochemicals, while the SDR2 and SDR5 oxidoreductases were MyA-specific (Figure S17A, Table 4). NAC102 was the only TF on the core list of 12 genes and is known to be a pivotal upstream component of other TFs in stress responses, including the detoxification programme and seed germination responses to flooding [56,62,63].

The ABA import carrier ABCG40 and the ACC transporter LHT1 were among those hormone transporters consistently up-regulated and down-regulated, respectively, by MyA and many of the other compounds (Table 2 and Table 4). The picture was far more diverse for the various auxin transporters for which both the expression patterns and polar localisation control tissue-specific auxin homeostasis and distribution [21,83,95,96,99]. The allelochemical NCS inhibits seed germination and seedling growth, and interference with seedling root auxin transport has been demonstrated to be one of its major mechanisms [6,20,29,30]. NCS modulated polar auxin transport in roots by interfering with subcellular trafficking and localisation of the AUX1, PIN2, PIN3, PIN4, and PIN7 proteins. While the expression of PIN1, PIN3 and PIN7 was down-regulated in seedling roots by NCS, and the expression of AUX1, PIN2 and PILS3 was up-regulated (Table 3). NCS also down-regulated the expression of the IAA transporters WAT1 and ABCB19, and up-regulated the expression of the IAA transporters ABCB4, ABCB11and ABCB21, and the IBA transporter ABCG36 which is closely related to ABCG37 (Table 3). Cao et al. [20] concluded that perturbation of auxin homeostasis by NCS was further affected by up-regulation of the IBA-conjugating UGT74E2, which was also observed upon MyA treatment (Table 2 and Table 4). The inhibition of root growth by NCS could however not be reverted by simultaneous IAA treatment [29], also not by a low IAA concentration which showed a partial reversion in the case of MyA (Figure 7A). Hu et al. [29] concluded that NCS acts on the auxin signalling pathway upstream of TIR1 via the degradation of AUX/IAA repressor proteins. Signalling via the TIR1-AUX/IAA pathway also seems to play an important role in auxin homeostasis and transport for MyA and other phytotoxins, but different AUX/IAA were regulated (down in most cases), and in several cases, in addition, ARF and WRKY genes were regulated (Table 3). Therefore, while the involvement of auxin signalling in the responses to different compounds appears to be a common theme, a diversity of TFs and auxin signalling factors seem to contribute to the regulation of auxin homeostasis and transport.

For the auxin transporters, MyA shares with NCS the down-regulation of PIN7 and up-regulation of ABCB4, ABCB11 (IAA transporter) and the IBA transporter ABCG37 (Figure 6C, Table 3). MyA differs in that AUX1 and PIN2 were slightly down-regulated (Figure 6A and Figure S15A) and not up-regulated as for NCS, and that WAT1 was up-regulated by MyA and down-regulated by NCS (Table 3). Among the PIN efflux carriers affected by phytotoxins down-regulation of PIN7 expression seems to be a primary target observed for MyA, NCS, citral, PAA_1_, FEN, and CMPP (Table 3), as well as for farnesene [7], rootin [114], weisiensin B [26], norhamane [28] and angelicin (Table 2). While *PIN7* was down-regulated in all these cases, for *PIN2* either up-regulation (NCS, naringenin, scutellarin, scutellarein, benzoic acid) or down-regulation (citral, farnesene, weisiensin B, norhamane) were reported [7,9,26,28,30,114,115]. For citral down-regulation of 6 *PIN* and 8 other auxin transporter genes was observed, while for trans-chalcone and BOA none of the *PIN* transporter genes were regulated, but *PILS* genes were up-regulated (Table 3). MyA treatment of cress germinating seeds did not affect *PILS* gene expression, but shared with trans-chalcone and BOA the up-regulation of the IBA transporter ABCG37 (Table 3). We conclude from these comparisons that all investigated compounds may interfere with auxin signalling and transporter gene expression potentially leading to altered localised auxin homeostasis and distribution. Which of the multi-molecular targets among the large and diverse group of auxin transporters differs is used to achieve this differs among the various compounds. The natural phytotoxin MyA achieves this by combining down-regulation of the *PIN7* gene, a widespread target, with up-regulation of *WAT1*, *ABCG37* and several *ABCB* genes, which are less widespread targets. Published [17,37,38] and new [this work] physiological, biochemical and transcriptome results, including the MyA-triggered detoxification response programme, support the conclusion that MyA exerts its phytotoxic activity on germinating seeds and growing seedlings by multiple molecular mechanisms.

## 3. Materials and Methods

### 3.1. Plant Material and Germination Assays

*Lepidium sativum* L. FR14 seeds (“Keimsprossen”, Juliwa) [38] were propagated, and harvested seeds were dried under 15% relative humidity before being stored at −20 °C until used in the experiment. About 30 to 50 seeds were plated in a petri dish (6 cm diameter) onto a filter paper (MN713, Macherey-Nagel, Düren, Germany) moistened with 1 mL of autoclaved deionised water or an aqueous solution of compounds at the indicated concentration, incubated in a growth chamber (MLR-352, Panasonic, Bracknell, UK) set at 20 °C with continuous light (approximately 100 µmol s^−1^ m^−2^). Testa and endosperm rupture was scored over time using a binocular microscope.

### 3.2. Chemicals

Myrigalone A, myrigalone B and myrigalone D, and 2′,4′-dihydroxy-6′-methoxy-3′5′-dimethylchalcone (DMC) were extracted from *Myrica gale* fruits and plants as described [36] by Syngenta’s Jealott’s Hill International Research Centre (Bracknell, UK). Gibberellin A_4+7_ and paclobutrazol were purchased from Duchefa Biochemie (Haarlem, The Netherlands). Phloretin, dihydrochalcone, naringenin, neohesperidin dihydrochalcone, daphnetin, psoralen, angelicin, ferulic acid, acacetin, carbonyl cyanide m-chlorophenyl hydrazone (CCCP), 2,3,5-triiodobenzoic acid (TIBA), indole-3-acetic acid (IAA), aminoethoxyvinylglycine (AVG), L-kynurenine, and hydrogen peroxide solution (30% (*w*/*w*)) were purchased from Sigma-Aldrich (St Louis, MO, USA). 5-(4-Chlorophenyl)-4*H*-1,2,4-triazole-3-thiol (yucasin) was purchased from Carbosynth Ltd. (Compton, Berkshire, UK). 4-(2,4-dimethylphenyl)-2-(1*H*-indol-3-yl)-4-oxobutanoic acid (auxinole) was purchased from Cambridge Bioscience (Cambridge, UK). All the compounds were dissolved in DMSO except for phloretin, dihydrochalcone, naringenin and neohesperidin dihydrochalcone which were dissolved in methanol. Controls were performed with basal solvent (0.1% (*v*/*v*) DMSO or methanol) as appropriate.

### 3.3. Plant Hormone Extraction and Quantification

For endogenous hormone and the related metabolite quantification, 5 replicates of approximately 30 seeds were incubated either with 0.1% (*v*/*v*) DMSO (control) or 0.5 mM MyA for the indicated period at 20 °C in continuous light and sampled. Sampled seeds were ground into fine powder in liquid nitrogen and lyophilised. Extraction and quantification were performed using 10 mg freeze-dried powder as described previously [93,116,117].

### 3.4. Extraction of RNA and RT-qPCR Analysis

Total RNA was extracted from the dry or imbibed seeds (approx. 20 mg dry weight) at the indicated time points using RNAqueous^TM^ columns and RNA isolation aid (Invitrogen, Waltham, MA, USA) as described previously [118] with DNase-I treatment (Qiagen, Manchester, UK) before the LiCl precipitation. The quality of extracted RNA was checked spectrophotometrically and by Bioanalyser analysis using the RNA 6000 nano assay (Agilent, Santa Clara, CA, USA). RNA samples with 260/280 ratios greater than 1.8, 260/230 ratios greater than 2.7, and RIN values greater than 8.0 were used for further analyses. For the RT-qPCR, cDNA was synthesised using Superscript Reverse transcriptase III (Invitrogen, Waltham, MA, USA) from 1 µg of total RNA in a volume of 20 µL according to the manufacturer’s instruction with a mixture of random pentadecamer primers, and qPCR was performed using ABsolute qPCR SYBR Green Mix (Thermo Scientific, Waltham, MA, USA) and a BioRad CFX96 thermal cycler (BioRad, Hercules, CA, USA) as described [119]. Candidate reference genes were tested and selected using geNorm software as described [120]. Primers for each gene were designed using Geneious software (ver. 8.1.9, Geneious, Auckland, New Zealand). Primer sequences for the target and reference genes are listed in Supplemental Table S1. Expression values were calculated using the 2^−ΔCt^ method [119] against the three best reference genes, *PP2A* (similar to At3g25800), *CAC AP2M* (similar to At5g46630) and *Hobbit* (similar to At2g20000).

### 3.5. RNAseq Analysis

Messenger RNA was enriched by polyA isolation using an NEBNext^®^ Poly(A) mRNA Magnetic Isolation Module (New England Biolabs (NEB), Ipswich, MA, USA). Libraries were prepared using NEBNext^®^ Ultra™ II Directional RNA Library Prep Kit (NEB) and a total of 30 libraries (5 replicates per samples) were sequenced using an Illumina HiSeq X platform (Illumina Inc., San Diego, CA, USA) generating ~26 million 150 bp paired-end reads per sample. Unitigs were assembled in ABySS [121] and collapsed into a single sequence set using CD-HIT-EST at 98% identity. Collapsed unitigs were assembled using MIRA [122], and the assembled contigs and unassembled unitigs were combined for scaffolding in ABySS. Only those transcripts with a minimum length of 200 bp were retained. Reads were mapped to the final assembly using BWA [123], and read counts were analysed using HTseq [124]. The similarity of all the samples was analysed by PCA. Differential expression analysis was performed using edgeR [42] or DESeq2 [43] based on the criteria of a log2 fold change greater than 1 (false discovery rate <0.05) using all the transcripts which had minimally 5 counts per million transcripts for 4 replications. Selected contig sequences were checked and verified using the *Lepidium sativum* genome data v1.1 produced by “Brassicales Map Alignment Project, DOE-JGI, http://bmap.jgi.doe.gov/” (accessed on 1 December 2021).

## Figures and Tables

**Table 1:** The number of differentially expressed transcript contigs from the pairwise comparison in the *Lepidium sativum* seed transcriptome datasets (see Data S1 and S2 for specific contig details).

Comparison	Up-Regulated Contigs in MyA Treatment	Down-Regulated Contigs in MyA Treatment
6 h MyA/6 h control	11 ^a^ (12) ^b^	3 (4)
12 h MyA/12 h control	180 (4723)	24 (27)
18 h MyA/12 h control	889 (1341)	419 (491)

^a^ Number of transcript contigs that showed BLAST matches against *Arabidopsis thaliana* or *Brassica* spp. databases. ^b^ Overall number of detected contigs are shown in parenthesis; these may also include potential contamination from other species as they did not provide BLAST matches to either *A. thaliana* or *Brassica* spp.

**Table 2:** Comparison of compound treatment on the expression of selected genes in germinating *Lepidium sativum* (*Lesa*) seeds. Normalised RT-qPCR ratios (compound/control at the times indicated) for myrigalone A (MyA), MyB, MyD and angelicin treatments. Mean ± SEM from RNA extracted from three biological replicate plates.

Gene Name	Control 12 h/Con 12 h	MyA ^b^ 12 h/Con 12 h	MyA ^b^ 18 h/Con 12 h	MyB ^b^ 12 h/Con 12 h	MyD ^b^ 12 h/Con 12 h	Angelicin ^c^ 12 h/Con 12 h	Angelicin ^c^ 18 h/Con 12 h
LesaGSTU25	1.0 ± 0.1	23.1 ± 12.7	49.0 ± 15.4	0.3 ± 0.2	2.5 ± 2.1	0.5 ± 0.3	0.2 ± 0.1
LesaNAC102	1.0 ± 0.1	14.6 ± 4.9	4.1 ± 0.7	0.3 ± 0.1	0.5 ± 0.3	0.4 ± 0.1	0.5 ± 0.1
LesaUGT73B5	1.0 ± 0.2	12.4 ± 3.9	8.7 ± 0.8	1.0 ± 0.2	1.2 ± 0.4	0.6 ± 0.1	0.6 ± 0.1
LesaERF2	1.0 ± 0.2	6.6 ± 2.8	0.5 ± 0.1	0.3 ± 0.2	2.0 ± 0.9	0.3 ± 0.2	0.2 ± 0.1
LesaABCG37	1.0 ± 0.2	5.2 ± 0.3	3.1 ± 0.3	0.9 ± 0.3	1.0 ± 0.2	0.5 ± 0.2	1.2 ± 0.4
LesaOXI1	1.0 ± 0.4	4.8 ± 2.3	0.8 ± 0.1	0.7 ± 0.5	0.8 ± 0.2	0.3 ± 0.1	0.3 ± 0.1
LesaCYP81D8	1.0 ± 0.3	4.4 ± 2.2	0.2 ± 0.1	0.2 ± 0.1	0.6 ± 0.3	0.4 ± 0.1	0.1 ± 0.0
LesaOPR1/2	1.0 ± 0.1	4.3 ± 1.1	4.4 ± 0.1	1.6 ± 0.5	1.4 ± 0.2	0.9 ± 0.1	1.0 ± 0.1
LesaWRKY75	1.0 ± 0.1	3.6 ± 1.1	2.9 ± 0.3	0.6 ± 0.2	0.6 ± 0.1	0.5 ± 0.1	0.8 ± 0.2
LesaPER13	1.0 ± 0.2	3.4 ± 0.5	1.1 ± 0.2	0.9 ± 0.1	0.7 ± 0.1	0.5 ± 0.1	0.6 ± 0.2
LesaWRKY23	1.0 ± 0.1	2.9 ± 1.5	0.9 ± 0.1	1.0 ± 0.5	1.7 ± 0.9	0.6 ± 0.0	0.6 ± 0.1
LesaNAC005	1.0 ± 0.5	2.8 ± 1.5	6.5 ± 1.8	1.8 ± 0.6	1.7 ± 0.3	1.0 ± 0.3	3.6 ± 0.7
LesaUGT74E2	1.0 ± 0.3	2.7 ± 0.7	0.7 ± 0.1	0.4 ± 0.3	0.4 ± 0.1	0.4 ± 0.1	0.1 ± 0.0
LesaABCB4	1.0 ± 0.2	2.3 ± 1.2	1.5 ± 0.2	0.8 ± 0.1	1.2 ± 0.2	0.8 ± 0.2	1.2 ± 0.3
LesaAOX1A	1.0 ± 0.1	2.2 ± 1.3	0.5 ± 0.1	0.2 ± 0.0	0.6 ± 0.4	0.3 ± 0.1	0.3 ± 0.2
LesaCYP81D4	1.0 ± 0.3	2.2 ± 0.2	0.4 ± 0.1	1.0 ± 0.4	0.9 ± 0.4	0.9 ± 0.2	0.2 ± 0.1
LesaUGT75B1	1.0 ± 0.3	2.1 ± 1	0.5 ± 0.1	1.5 ± 1.0	0.9 ± 0.2	0.4 ± 0.0	0.4 ± 0.2
LesaUGT75D1	1.0 ± 0.0	1.9 ± 0.2	1.6 ± 0.1	0.4 ± 0.1	0.7 ± 0.2	0.6 ± 0.1	1.1 ± 0.5
Monooxygenase (AT5G64250) ^a^	1.0 ± 0.1	1.7 ± 0.2	0.9 ± 0.1	0.8 ± 0.1	1.0 ± 0.2	0.9 ± 0.1	0.4 ± 0.1
LesaABCG34	1.0 ± 0.2	1.6 ± 0.4	1.5 ± 0.2	0.8 ± 0.3	0.8 ± 0.2	0.9 ± 0.1	1.5 ± 0.8
LesaGSTU19	1.0 ± 0.1	1.5 ± 0.1	0.6 ± 0.1	0.9 ± 0.3	0.9 ± 0.3	0.8 ± 0.1	0.3 ± 0.1
Oxidoreductase (AT3G44190) ^a^	1.0 ± 0.1	1.5 ± 0.2	4.3 ± 0.7	1.1 ± 0.0	1.3 ± 0.2	1.1 ± 0.2	0.7 ± 0.1
LesaFSD1	1.0 ± 0.0	1.5 ± 0.1	5.5 ± 1.6	0.6 ± 0.1	0.7 ± 0.3	0.5 ± 0.1	4.1 ± 1.0
LesaPER70	1.0 ± 0.4	0.6 ± 0.2	0.4 ± 0.1	1.1 ± 0.2	0.6 ± 0.2	0.3 ± 0.1	0.7 ± 0.2
LesaTAT2	1.0 ± 0.1	0.6 ± 0.1	0.4 ± 0.0	0.4 ± 0.1	0.9 ± 0.3	0.7 ± 0.1	0.5 ± 0.2
LesaPER45	1.0 ± 0.5	0.6 ± 0.3	1.6 ± 0.6	0.3 ± 0.2	0.9 ± 0.3	0.3 ± 0.1	3.2 ± 1.0
LesaARF11	1.0 ± 0.3	0.5 ± 0.2	0.7 ± 0.2	1.4 ± 0.5	1.2 ± 0.2	0.9 ± 0.2	1.1 ± 0.4
LesaCYP78A7	1.0 ± 0.1	0.4 ± 0.1	0.2 ± 0.0	0.8 ± 0.1	0.7 ± 0.1	0.9 ± 0.2	0.3 ± 0.1
LesaUMAMIT25	1.0 ± 0.2	0.4 ± 0.1	0.3 ± 0.0	0.5 ± 0.1	0.6 ± 0.2	0.3 ± 0.1	0.4 ± 0.2
LesaPIN7	1.0 ± 0.1	0.4 ± 0.1	0.3 ± 0.1	0.5 ± 0.2	0.6 ± 0.2	0.5 ± 0.1	0.7 ± 0.3
LesaLHT1	1.0 ± 0.2	0.3 ± 0.1	0.3 ± 0.0	0.5 ± 0.0	0.6 ± 0.2	0.3 ± 0.1	1.1 ± 0.2
LesaSKS15	1.0 ± 0.0	0.3 ± 0.2	2.2 ± 0.7	0.6 ± 0.1	0.9 ± 0.3	0.6 ± 0.2	3.5 ± 0.4

^a^ putative *Lesa* orthologs of the AGIs: monooxygenase (AT5G64250), oxidoreductase (AT3G44190); ^b^ 0.5 mM; ^c^ 0.1 mM.

**Table 3:** Comparison of compound treatment on the expression of auxin signalling and hormone transporter genes. Results from myrigalone A (MyA) treated germinating *Lepidium sativum* seeds were compared to seeds, seedlings or root cultures of *Arabidopsis thaliana* treated with the allelochemicals *trans*-chalcone (*t*CHC), narciclasine (NCS), citral, benzoxazolin-2(*3H*)-one (BOA), or with the oxylipin A_1_-phytoprostane (PPA_1_), the herbicide safeners fenclorim (FEN) or CMMP, or with methotrexate (MTX) or 2,4-dinitrophenol (DNP). Transcript abundance ratios compound/control ≥2 or ≤0.5 were considered as UP or DOWN (DN), respectively, or otherwise labelled as not at least 2-fold regulated (“-”). See Figures S1 and S2 for chemical structure. Published transcriptomes: *t*CHC [19], citral [18], BOA [4], PPA1 [48], FEN and CMMP [13], MTX and DNP [31], and NCS root transcriptome [20] and NCS (marked with “*”) root RT-qPCR [30]. For these transcriptomes, published lists of at least 2-fold regulated DEGs were used; for FEN and CMMP, the lists used were at least 2-fold regulated DEGs. “n.a.”, gene not available in sequenced transcript contigs.

		Ratio MyA/Con		Ratio Compound/Control: ≥2 (UP), ≤2 (DN; Down) or Not 2-Fold Regulated (“-”)											
		Myrigalone A		MyA	*t*CHC	*t*CHC	NCS	Citral	Citral	BOA	PAA_1_	FEN	CMPP	MTX	DNP
AGI	Gene Name	12/12 h	18/12 h	Seeds	Roots	Shoots	Roots	Roots	Shoots	Seedlings	Seedlings	Root	Root	Seeds	Seeds
												Cult.	Cult.		
**Selected hormone transporter genes (known hormone specificity)**															
AT1G15520	ABCG40 (ABA)	4.3	4.6	UP	UP	UP	-	UP	UP	UP	UP	-	UP	-	-
AT1G71960	ABCG25 (ABA)	0.9	0.8	-	-	-	-	-	-	-	-	-	-	-	-
AT1G31770	ABCG14 (CK)	0.9	3.6	UP	-	-	-	DN	-	-	-	-	-	-	DN
AT4G39850	ABCD1/CTS (OPDA)	0.9	0.9	-	-	-	-	DN	-	-	-	-	-	-	-
AT3G55090	ABCG16 (JA)	n.a.	n.a.	n.a.	UP	-	-	UP	-	-	UP	-	-	UP	-
AT2G26690	NPF6.2 (GA)	1.0	4.9	UP	-	-	DN	-	DN	-	-	-	-	DN	DN
AT1G12110	NPF6.3 (GA)	1.0	3.2	UP	DN	-	-	DN	-	-	-	-	DN	-	-
AT5G40780	LHT1 (ACC)	0.5	0.8	DN	DN	-	-	DN	-	-	-	-	-	DN	DN
**Auxin transporter: IAA influx (AUX1) and efflux (PIN, PILS) carrier**															
AT2G38120	AUX1	0.8	0.8	-	-	-	UP *	-	-	-	DN	-	DN	-	-
AT1G73590	PIN1	0.8	0.7	-	-	-	DN *	DN	-	-	DN	-	-	-	-
AT5g57090	PIN2	0.8	0.7	-	-	-	UP *	DN	-	-	-	-	-	-	-
AT1G70940	PIN3	0.8	1.3	-	-	-	DN *	-	DN	-	-	DN	-	-	-
AT2G01420	PIN4	0.9	1.2	-	-	-	DN	-	DN	-	-	-	-	-	DN
AT5G16530	PIN5	n.a.	n.a.	n.a.	-	-	-	-	-	-	-	-	-	-	-
AT1G77110	PIN6	0.9	0.8	-	-	-	-	-	DN	-	-	-	-	DN	-
AT1G23080	PIN7	0.5	0.6	DN	-	-	DN	-	DN	-	DN	DN	DN	-	-
AT5G15100	PIN8	n.a.	n.a.	n.a.	-	-	-	-	-	-	-	-	-	-	DN
AT1G20925	PILS1	n.a.	n.a.	n.a.	-	-	-	-	-	-	-	-	-	-	-
AT1G71090	PILS2	0.9	1.1	-	-	-	-	DN	-	-	-	-	-	-	-
AT1G76520	PILS3	1.3	1.3	-	UP	-	UP	-	-	UP	-	UP	UP	-	-
AT1G76530	PILS4	n.a.	n.a.	n.a.	-	UP	-	-	-	-	-	-	-	UP	UP
AT2G17500	PILS5	1.2	1.4	-	UP	UP	-	UP	-	-	-	UP	-	-	-
AT5G01990	PILS6	0.9	0.8	-	-	-	-	DN	-	-	-	-	-	-	-
AT5G65980	PILS7	0.9	1.2	-	-	-	-	-	-	-	-	-	-	-	-
**Auxin transporter: ABC transporter and UMAMIT15 (WAT1) auxin carrier (known auxin specificity)**															
AT1G75500	WAT1 (IAA)	0.9	4.4	UP	-	-	DN	-	-	-	DN	-	DN	-	-
AT2G36910	ABCB1 (IAA)	0.7	0.9	-	-	-	-	-	-	-	-	-	-	-	-
AT2G47000	ABCB4 (IAA)	3.8	4.8	UP	-	-	UP	-	-	-	UP	UP	UP	-	-
AT1G02520	ABCB11	2.1	1.9	UP	-	UP	UP	DN	-	-	-	-	-	-	-
AT1G28010	ABCB14 (cytokinin)	1.7	7.1	UP	-	-	-	-	DN	-	-	-	-	DN	DN
AT3G28345	ABCB15	1.1	0.8	-	-	-	-	DN	-	-	-	-	-	UP	-
AT3G28860	ABCB19 (IAA)	0.8	0.8	-	-	-	DN	-	DN	-	-	-	-	-	-
AT3G62150	ABCB21 (IAA)	1.0	0.9	-	-	-	UP	-	DN	-	-	-	-	-	-
AT3G53480	ABCG37 (IBA)	4.6	5.1	UP	-	UP	-	-	-	UP	-	-	-	-	-
AT1G59870	ABCG36 (IBA)	1.1	0.9	-	-	-	UP	DN	-	-	-	-	-	-	-
AT2G39350	ABCG1	1.2	1.8	-	-	-	UP	-	-	-	-	UP	UP	-	-
**Selected auxin signalling genes**															
AT3G62980	TIR1	0.9	0.8	-	-	-	-	-	DN	-	-	-	-	-	-
AT1G04240	IAA3/SHY2	0.8	4.1	UP	-	-	-	-	DN	-	DN	DN	DN	UP	-
AT1G04250	IAA17/AXR3	1.0	3.4	UP	-	DN	DN	DN	-	-	-	-	-	DN	-
AT2G22670	IAA8	0.9	1.2	-	-	-	DN	-	DN	-	-	-	-	-	-
AT5G57420	IAA33	1.2	0.4	DN	-	-	-	DN	-	-	-	-	-	-	-
AT1G59750	ARF1	0.8	0.5	DN	-	-	-	-	DN	-	-	-	-	-	-
AT2G46530	ARF11	0.3	0.4	DN	-	-	-	-	DN	-	-	-	-	-	-
AT3G61830	ARF18	0.8	0.5	DN	-	-	-	-	-	-	-	-	-	-	-
AT5G13080	WRKY75	4.5	5.7	UP	-	-	-	UP	-	-	UP	UP	UP	DN	UP
AT3G56710	SIB1	0.6	0.4	DN	-	-	-	-	-	UP	-	UP	UP	-	-
AT2G41180	SIB2	0.8	0.4	DN	-	UP	-	-	DN	-	-	-	-	-	-
AT2G47260	WRKY23	3.1	3.0	UP	-	-	-	DN	-	-	-	-	-	-	-
AT1G62300	WRKY6	2.1	2.4	UP	-	-	UP	DN	-	UP	-	UP	UP	-	-

**Table 4:** Comparison of compound treatment on the expression of selected detoxification, transporter and other genes. Results from myrigalone A (MyA) treated germinating Lepidium sativum seeds were compared to seeds, seedlings or roots cultures of *Arabidopsis thaliana* treated with *t*CHC, NCS, citral, BOA, PPA1, FEN, CMMP, MTX, DNP. Transcript abundance ratios compound/control ≥2 or ≤0.5 were considered as UP or DOWN (DN), respectively, or otherwise labelled as not at least 2-fold regulated (“-”). See Table 3 for abbreviations, references, and further details.

		Ratio MyA/Con		Ratio Compound/Control: ≥2 (UP), ≤2 (DN; Down) or Not 2-Fold Regulated (“-”)											
		Myrigalone A		MyA	*t*CHC	*t*CHC	NCS	Citral	Citral	BOA	PAA_1_	FEN	CMPP	MTX	DNP
AGI	Gene Name	12/12 h	18/12 h	Seeds	Roots	Shoots	Roots	Roots	Shoots	Seedlings	Seedlings	Root	Root	Seeds	Seeds
												Cult.	Cult.		
**GST, peroxidase, glutathione and ascorbate system genes (substrate)**															
AT1G17180	GSTU25	7.5	35.3	UP	UP	UP	UP	-	-	UP	UP	UP	-	-	-
AT1G78380	GSTU19 (OPDA)	2.5	2.0	UP	UP	UP	-	-	UP	-	UP	UP	UP	-	UP
AT1G78340	GSTU22	3.6	8.8	UP	UP	UP	UP	DN	-	UP	UP	-	UP	-	-
AT2G29490	GSTU1	5.2	7.1	UP	UP	UP	UP	DN	UP	UP	-	UP	UP	-	UP
AT3G09270	GSTU8	17.2	30.9	UP	-	UP	-	DN	-	UP	-	UP	UP	DN	DN
AT2G31570	GPX2	1.0	2.6	UP	-	-	-	DN	-	-	-	-	-	-	-
AT1G75270	DHAR2	4.9	8.0	UP	UP	UP	-	-	UP	-	UP	UP	UP	-	UP
AT1G07890	APX1	1.0	2.4	UP	-	-	-	DN	-	-	-	-	-	-	DN
AT4G35970	APX5	1.1	4.8	UP	-	-	-	DN	-	-	-	-	-	DN	DN
AT1G77100	PER13	2.6	1.7	UP	-	-	-	-	-	-	-	-	-	-	-
AT4G30170	PER45	0.7	5.0	UP	DN	-	DN	DN	-	DN	-	-	DN	DN	DN
AT4G25100	FSD1	1.0	6.9	UP	-	-	-	DN	DN	-	-	-	-	-	-
AT3G22370	AOX1A	2.3	3.3	UP	-	-	UP	-	-	-	-	UP	UP	UP	UP
**CYP750 and UGT protein genes (substrate)**															
AT4G37330	CYP81D4	2.2	0.9	UP	-	-	-	-	-	-	-	-	-	-	-
AT4G37370	CYP81D8	5.3	5.6	UP	UP	-	UP	UP	UP	UP	-	UP	UP	UP	UP
AT3G26290	CYP71B26	0.9	3.1	UP	-	DN	-	-	-	-	-	-	-	-	-
AT3G20950	CYP75A32	1.2	5.3	UP	-	-	-	DN	-	-	-	-	-	UP	DN
AT2G45570	CYP76C2	2.5	3.7	UP	-	-	-	-	UP	-	-	-	-	DN	DN
AT4G15550	UGT75D1 (IBA)	1.6	2.7	UP	-	UP	-	-	UP	-	UP	UP	-	-	UP
AT1G05680	UGT74E2 (IBA)	2.4	3.1	UP	-	-	UP	UP	UP	UP	-	UP	UP	UP	UP
AT1G05560	UGT75B1 (IBA)	2.6	2.2	UP	-	UP	UP	UP	UP	UP	UP	UP	-	-	UP
AT2G15480	UGT73B5	10.3	13.1	UP	-	UP	UP	UP	UP	UP	-	UP	UP	-	UP
AT4G34138	UGT73B1	1.7	2.3	UP	-	UP	-	-	UP	UP	-	UP	UP	DN	UP
**MATE, ABC, UMAMIT and aquaporin transporter** (* see Table 3 for hormone-specific ABC transporter)															
AT1G71140	DTX14	3.2	1.4	UP	UP	-	UP	UP	-	-	UP	-	UP	-	UP
AT4G25640	DTX35	1.3	2.1	UP	-	UP	-	-	-	-	-	UP	UP	-	-
AT1G66760	DTX9	1.1	2.9	UP	UP	-	-	-	DN	-	-	-	-	-	-
AT1G33100	DTX2	1.8	2.8	UP	-	-	-	DN	-	-	-	-	-	-	-
AT5G52450	DTX16	1.1	3.0	UP	-	-	UP	DN	-	-	-	-	-	-	-
AT2G36380	ABCG34 *	1.5	2.7	UP	-	UP	-	DN	-	-	-	-	-	-	UP
AT4G01450	UMAMIT30	1.4	2.7	UP	-	-	DN	DN	-	-	-	-	-	-	-
AT4G28040	UMAMIT33	1.1	3.3	UP	UP	UP	DN	DN	DN	-	-	-	-	DN	-
AT4G30420	UMAMIT34	2.2	8.8	UP	-	-	-	DN	-	-	-	-	-	-	-
AT3G56620	UMAMIT10	1.6	5.1	UP	-	-	-	DN	-	-	-	-	-	UP	-
AT3G26520	TIP1;2	0.9	2.1	UP	-	-	DN	DN	DN	-	-	-	-	DN	DN
AT1G73190	TIP3;1	2.7	1.4	UP	-	-	-	-	-	-	-	-	-	UP	UP
AT1G01620	PIP1;3	0.9	2.1	UP	-	-	DN	DN	-	-	-	-	-	-	-
AT2G16850	PIP2;8	1.0	3.4	UP	-	-	-	-	DN	-	-	-	-	-	-
AT2G37170	PIP2;2	0.8	2.2	UP	DN	-	DN	DN	-	-	-	-	-	-	-
AT2G39010	PIP2;6	1.7	4.2	UP	-	-	DN	-	DN	-	-	-	-	DN	-
**Transcription factors**															
AT1G02250	NAC005	3.9	9.5	UP	-	-	-	DN	-	-	-	-	-	-	-
AT1G77450	NAC032	3.1	2.8	UP	UP	UP	-	DN	UP	-	-	UP	UP	-	UP
AT5G08790	NAC081/ATAF2	2.1	1.5	UP	UP	-	UP	UP	-	-	-	-	-	-	-
AT5G63790	NAC102	3.8	3.9	UP	UP	UP	UP	-	UP	UP	UP	-	UP	-	UP
AT1G71520	ERF20	12.4	8.3	UP	UP	-	UP	UP	UP	-	-	-	-	-	-
AT1G74930	ERF18/ORA47	7.0	4.0	UP	-	-	DN	-	DN	-	-	-	-	-	-
AT3G23230	ERF98/TDR1	5.1	8.3	UP	-	-	-	-	-	-	-	-	-	-	-
AT5G47220	ERF2	2.9	1.8	UP	-	-	-	-	DN	-	-	-	-	-	UP
**Other genes**															
AT1G76680	OPR1/2	8.0	9.9	UP	UP	-	UP	UP	UP	UP	UP	UP	UP	-	UP
AT5G22140	Oxidoreductase	27.9	49.4	UP	UP	-	UP	UP	UP	-	-	-	-	-	-
AT3G44190	Oxidoreductase	91	349	UP	UP	-	-	-	UP	-	-	UP	-	-	-
AT4G13180	Oxidoreductase	6.4	43.5	UP	UP	UP	-	-	-	UP	UP	UP	UP	DN	UP
AT1G55920	SAT1	4.7	5.6	UP	UP	-	UP	-	UP	UP	UP	UP	UP	-	UP
AT5G39050	PMAT1	2.0	2.6	UP	UP	UP	UP	UP	UP	UP	-	UP	UP	-	UP
AT4G01870	tolB	6.7	5.3	UP	UP	-	UP	UP	UP	UP	UP	UP	UP	-	UP
AT4G24160	α/β-hydrolase	2.0	1.5	UP	UP	-	UP	-	UP	UP	-	UP	UP	-	UP
AT1G64670	BDG1 (α/β-hyd.)	1.0	3.0	-	-	-	-	-	-	-	-	-	-	-	-
AT1G06570	HPPD	1.1	0.9	-	UP	-	-	-	DN	-	-	-	-	-	UP

## Data Availability

The RNAseq data discussed in this publication have been deposited in NCBI’s Gene Expression Omnibus [125] (Edgar et al., 2002) and are accessible through GEO Series accession number GSE200989 (https://www.ncbi.nlm.nih.gov/geo/query/acc.cgi?acc=GSE200989 (accessed on 24 March 2022)). All other data presented or analysed in this published article are available online through the supplements and figshare https://doi.org/10.17637/rh.19586092 (accessed on 24 March 2022).

## Supplementary Materials

The following supporting information can be downloaded at: https://www.mdpi.com/article/10.3390/ijms23094618/s1.

## Abbreviations

ABI3, ABA insensitive3; ARF, auxin response factor; AUX1/LAX, AUXIN1/LIKE-AUX1 IAA influx carriers; BDG1, bodyguard1; CAX6, cation exchanger6; DEG, differentially regulated gene; FFT, flower flavonoid transporter; FPKM, fragments per kilobase per million; HIPP, heavy metal-associated isoprenylated plant protein; HPPD, p-hydroxyphenylpyruvate dioxygenase; IAA, indole-3-acetic acid; IBA, indole-3-butyric acid; OPT3, oligoPeptide transporter3; PCA, principal component analysis; PCY, plantacyanin; PHT, phosphate transporter; PIN, PIN-formed IAA efflux carrier; PILS, PIN-Likes auxin carrier; PMAT1, phenolic glucoside malonylTransferase1; SAT1, serine acetylTransferase1; SIB, sigma factor binding protein; TIR1, transport inhibitor response1; TF, transcription factor; UMAMIT, usually multiple amino acids move out transporters; WAT1, walls are thin1.
