# Supplementary material for: The Phytotoxin Myrigalone A Triggers a Phased Detoxification Programme and Inhibits Lepidium sativum Seed Germination via Multiple Mechanisms including Interference with Auxin Homeostasis
Source: Int J Mol Sci. 2022 Apr 21;23(9):4618. doi: 10.3390/ijms23094618 (PMC9104956; doi:10.3390/ijms23094618)
Supplement: Supplementary file 1 [file ijms-23-04618-s001.zip › Nakabayashi2022-MyA-supplements.pdf]

Supplementry Figures

Figure S1

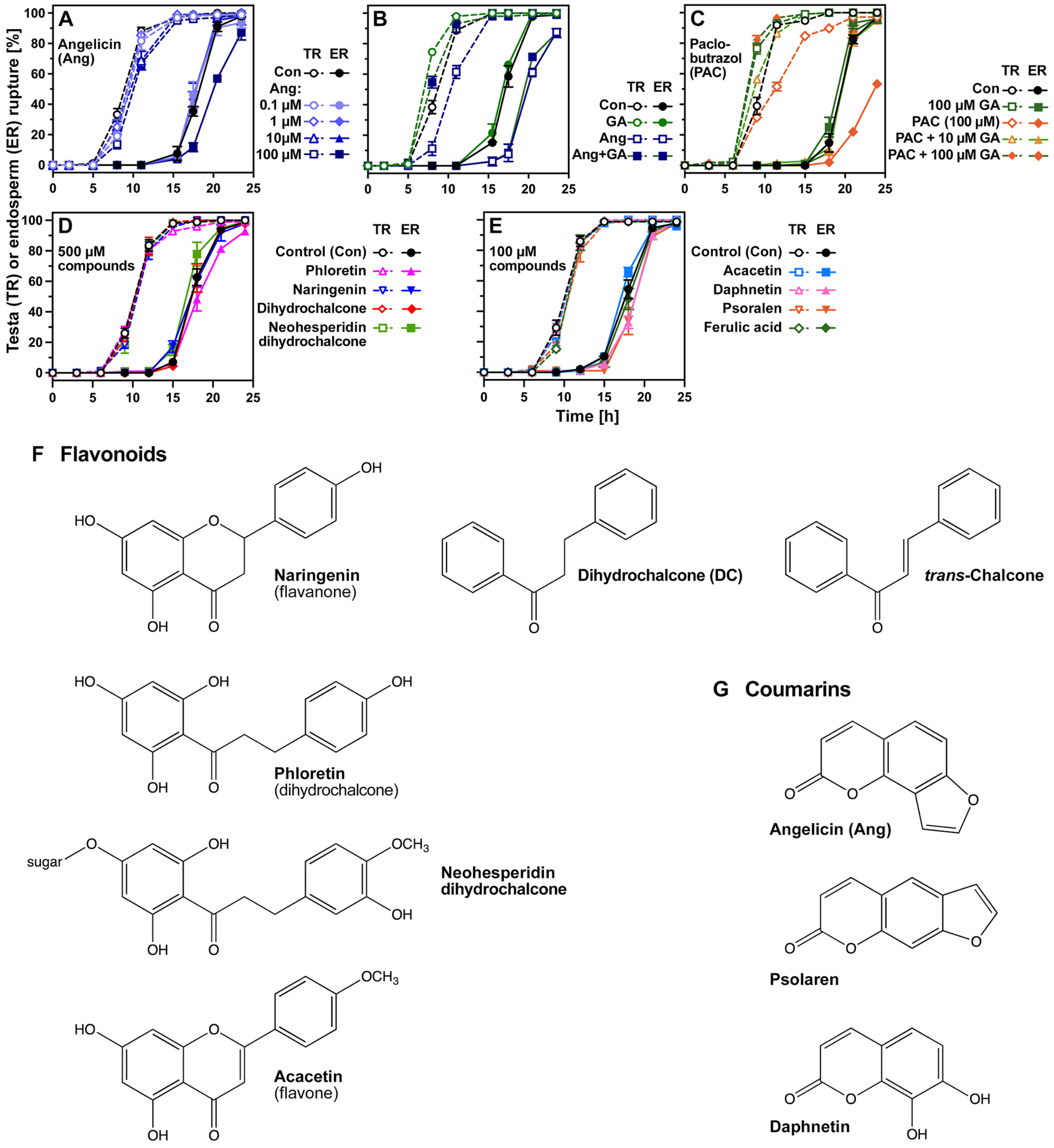

Figure S1. The effects of various compounds on *Lepidium sativum* seed germination. (**A**) The effect of the coumarin angelicin (Ang) on the kinetics of testa rupture (TR) and subsequent endosperm rupture (ER) without (Con, control) or with angelicin added at the concentrations indicated. (**B**) The effects of GA (100 µM GA_{4+7}) and angelicin (100 µM). (**C**) The effect of the GA biosynthesis inhibitor paclobutrazol (PAC). (**D**) The effects of naringenin and three dihydrochalcones. (**E**) The effects of acacetin, two coumarins and ferulic acid. (**F**) Chemical structures of the flavonoid compounds investigated. (**D**) Chemical structures of the coumarin compounds investigated. Seeds were incubated at 20 ºC in continuous white light, mean values ± SEM for 3 replicates each with ca. 30 seeds are shown.

Figure S2

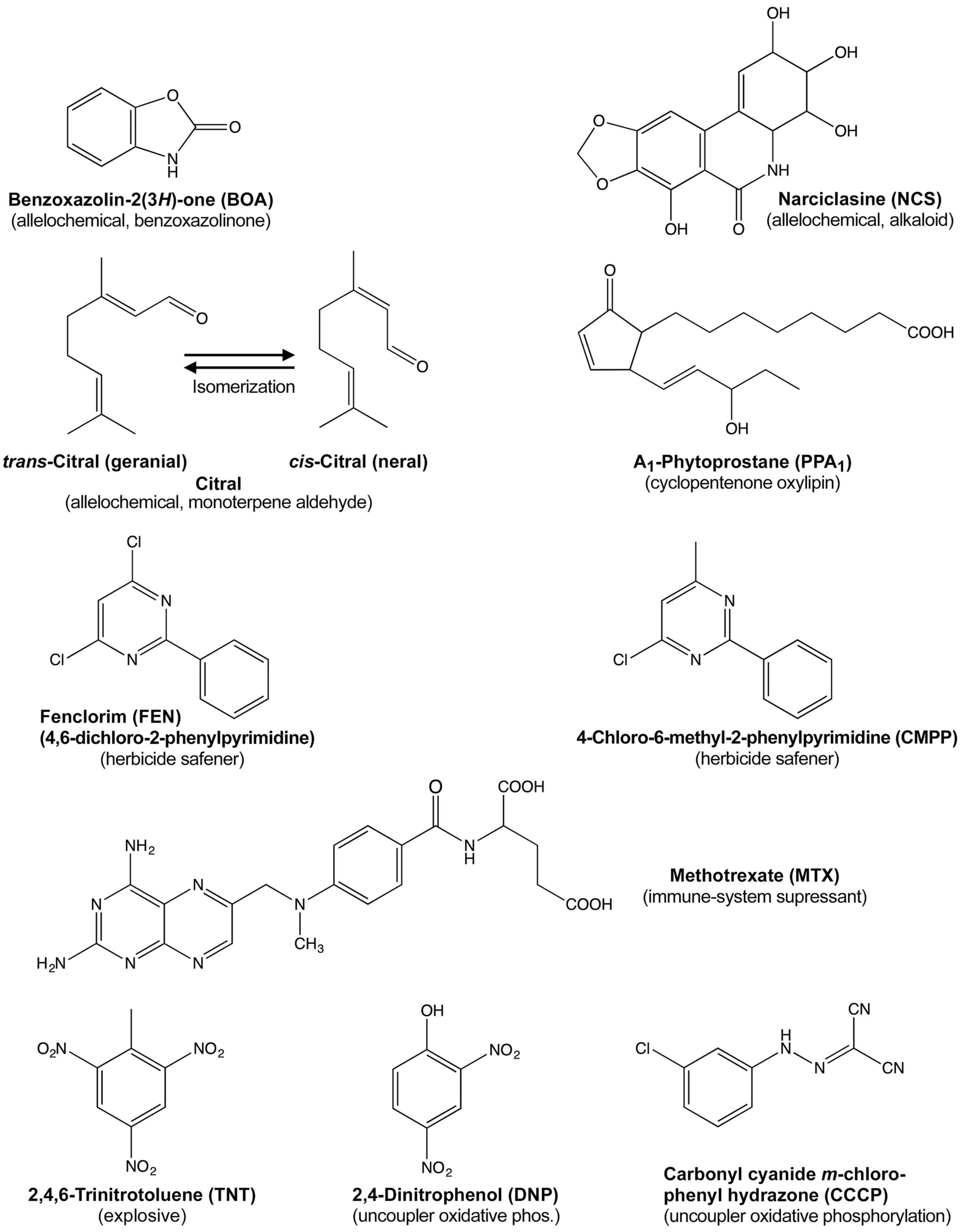

Figure S2. Chemical structures of the compounds used for comparing treatment transcriptome responses with *Arabidopsis thaliana*. Data mining of these transcriptomes [4, 8, 13, 18, 20, 31, 48] of seedlings (BOA, PAA_1, TNT), seedling roots (NCS, citral), seedling shoots (citral), root cultures (FEN, CMPP), or imbibed seeds (MTX, DNP) was used for the comparisons with myrigalone A (MyA) in Tables 3 and 4. This also included seedling root and shoot transcriptomes upon *trans*-chalcone treatment (see Figure S1) [19].

Figure S3

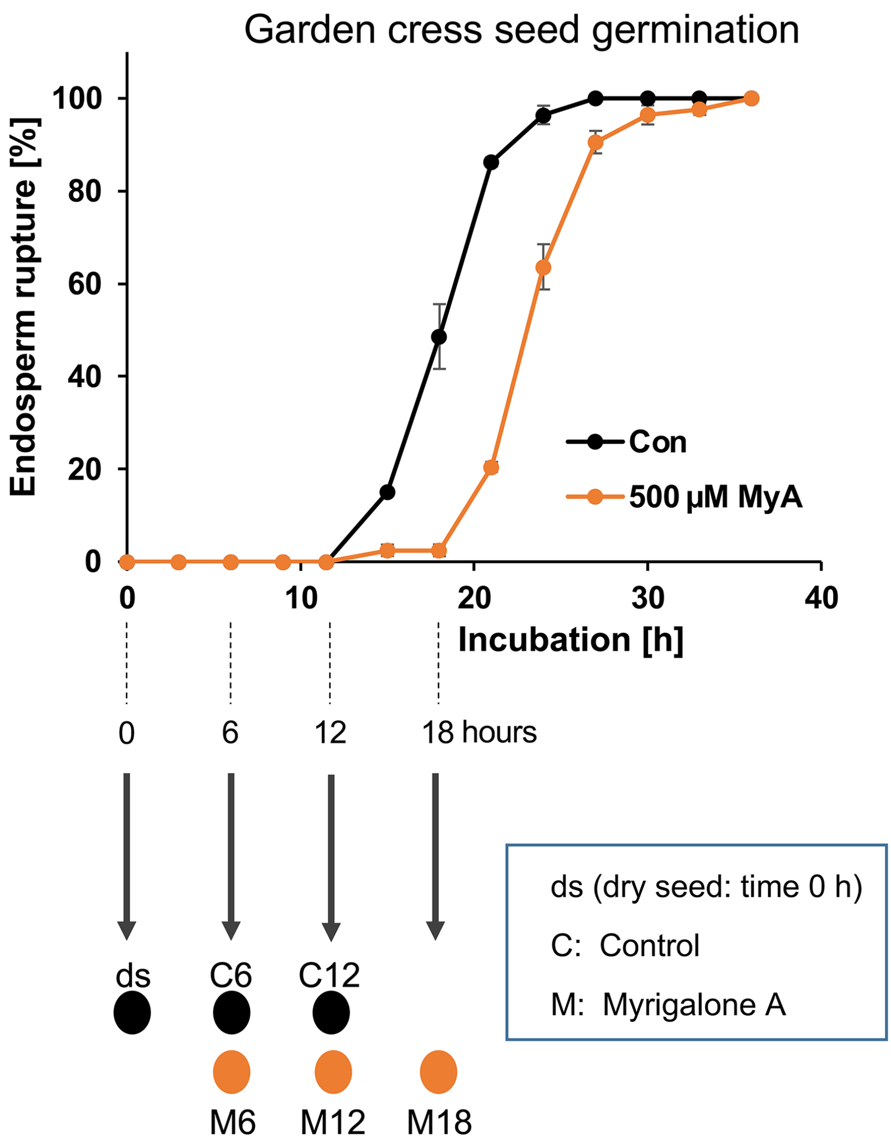

Figure S3. Schematic representation of sampling points for the hormone and RNAseq analyses during *Lepidium sativum* seed germination. Samples were prepared from dry seed (time 0 h) and imbibed seed at 6 h (midpoint until the start of endosperm rupture (ER) in control seed populations), 12 h (start of ER in control seed populations), 18 h (start of ER in MyA-treated seed populations).

Figure S4

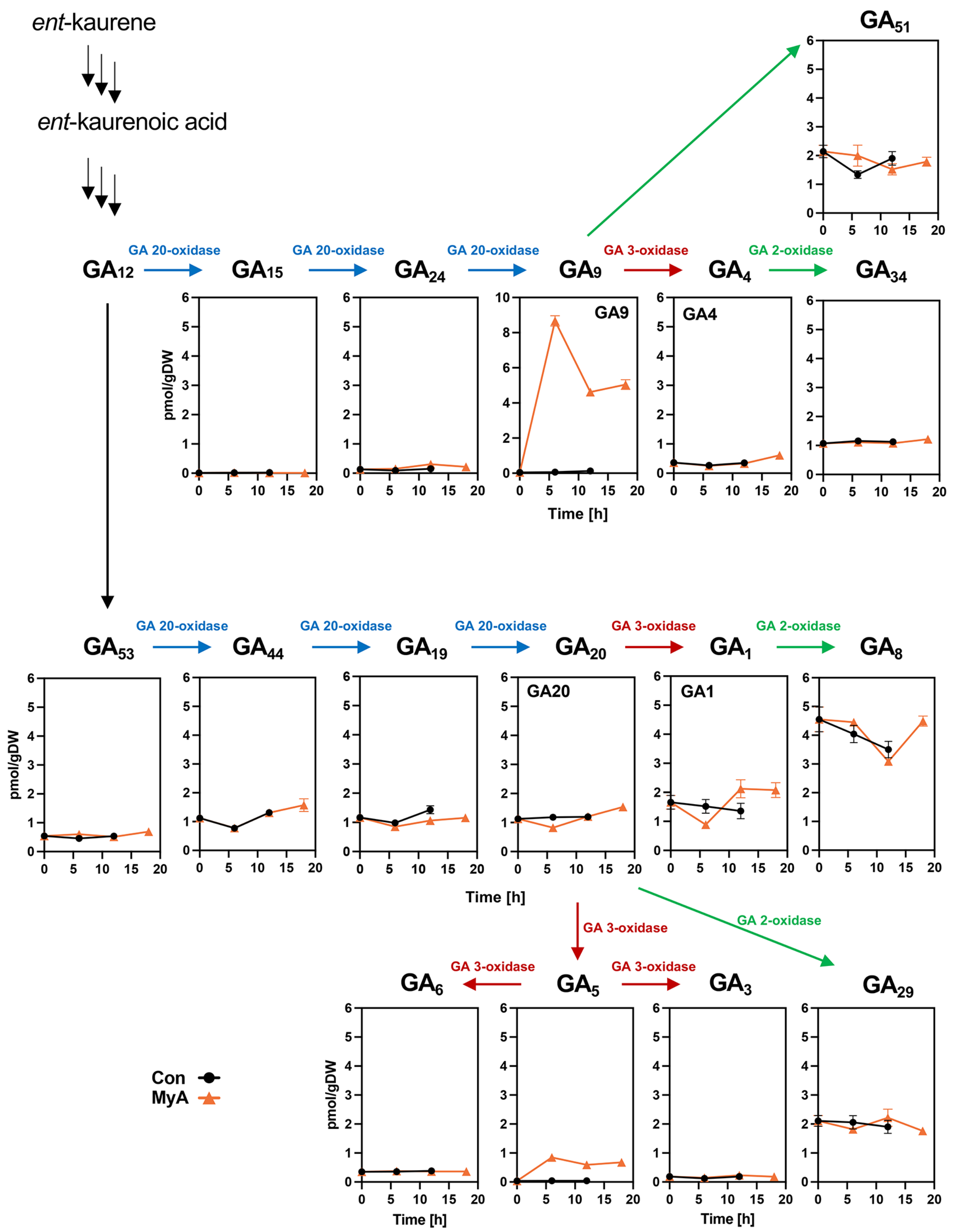

Figure S4. Gibberellin (GA)-related metabolite profiling of *Lepidium sativum* seed germination in response to 0.5 mM MyA. The results are presented in the frame of the GA metabolic pathway (key enzymes indicated) and as temporal patterns of endogenous metabolite contents (pmol/g dry weight) in whole seeds. Seeds were incubated at 20 ºC in continuous white light. Mean ± SEM values of five biological replicates.

Figure S5

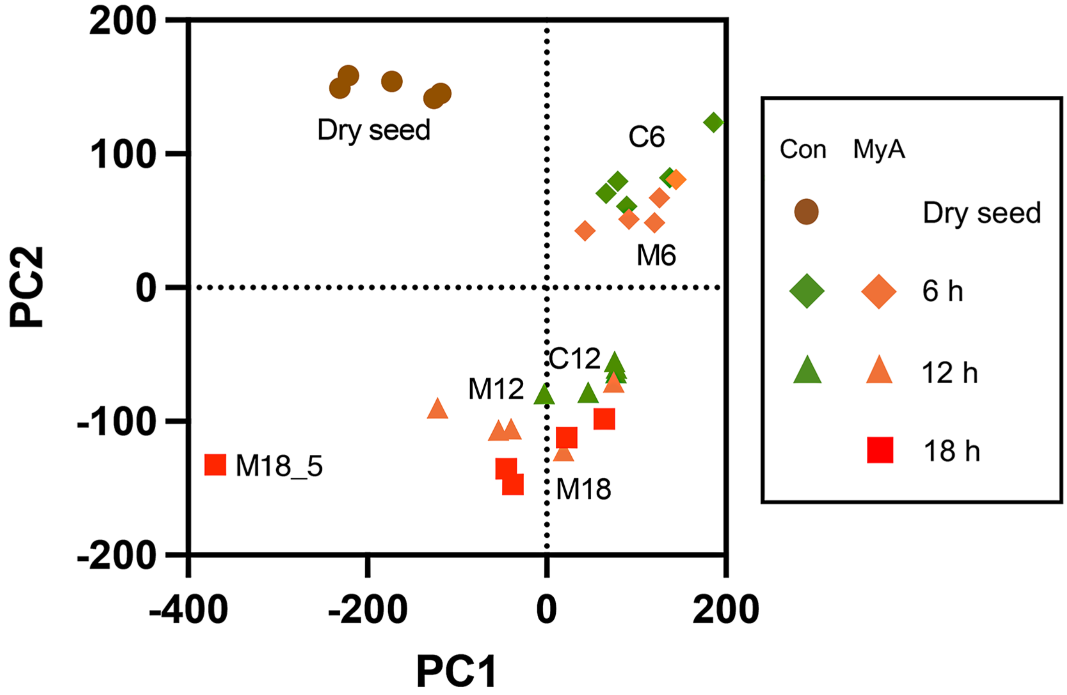

Figure S5. Principal Component Analysis (PCA) of transcriptome similarities of all samples. The PCA analysis revealed that the 5th MyA replicate at 18 h (M18-5) was an outlier, and it was therefore excluded from the further analysis. The other replicates clustered together in that the principal components PC1 and PC2 accounted for 37% and 24 % of the observed variance.

Figure S6

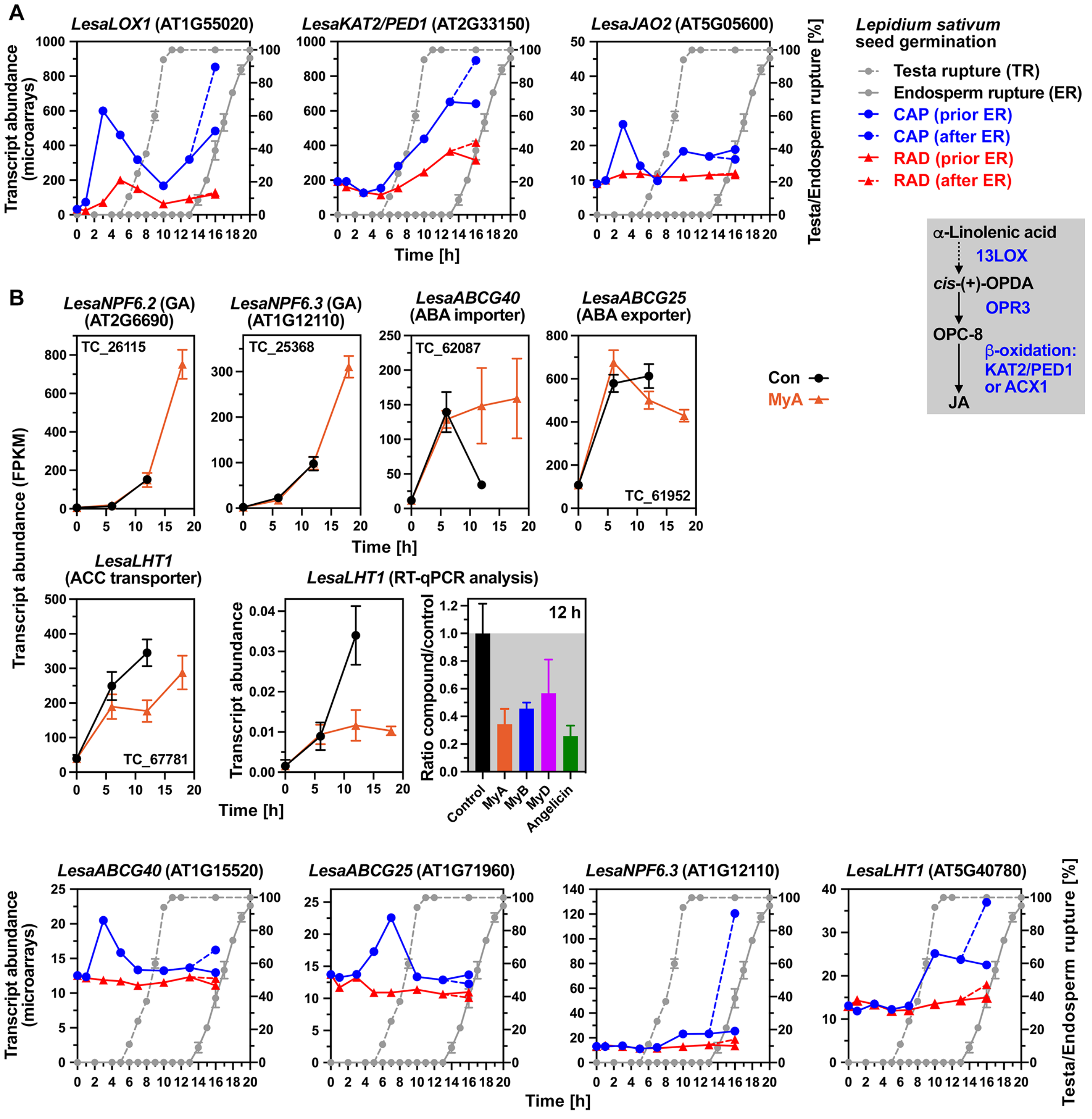

Figure S6. The effect of myrigalone A (MyA) and other compounds on the expression patterns of hormone biosynthesis and transporter genes during *Lepidium sativum* (cress) seed germination. (**A**) Spatiotemporal expression pattern of OPDA and jasmonate biosynthetic enzymes during cress seed germination. Transcript abundance mean ± SEM values in the CAP (micropylar endosperm) and RAD (radicle plus lower hypocotyl) compartments during cress seed germination derived from microarrays [40]. (**B**) The effect of MyA treatment (0.5 mM) on the transcript abundance patterns in germinating cress seeds. The names of *L. sativum* (*Lesa*) genes and the corresponding *A. thaliana* orthologs (AGI in brackets) are provided; see abbreviations for full names of genes. Transcript abundance patterns are presented as mean ± SEM values (relevant transcript contigs (TC-IDs) included in each graph) based on 4-5 (FPKM) and 3 (qRT-PCR) biological replicates. Relative transcript abundance ratios (compound/control) at 12 h during cress seed germination treated with MyA, MyB, MyD and angelicin were obtained by RT-qPCR analysis. Spatiotemporal expression pattern of hormone transporter genes in the CAP and RAD compartments during cress seed germination derived from microarrays [40].

Figure S7

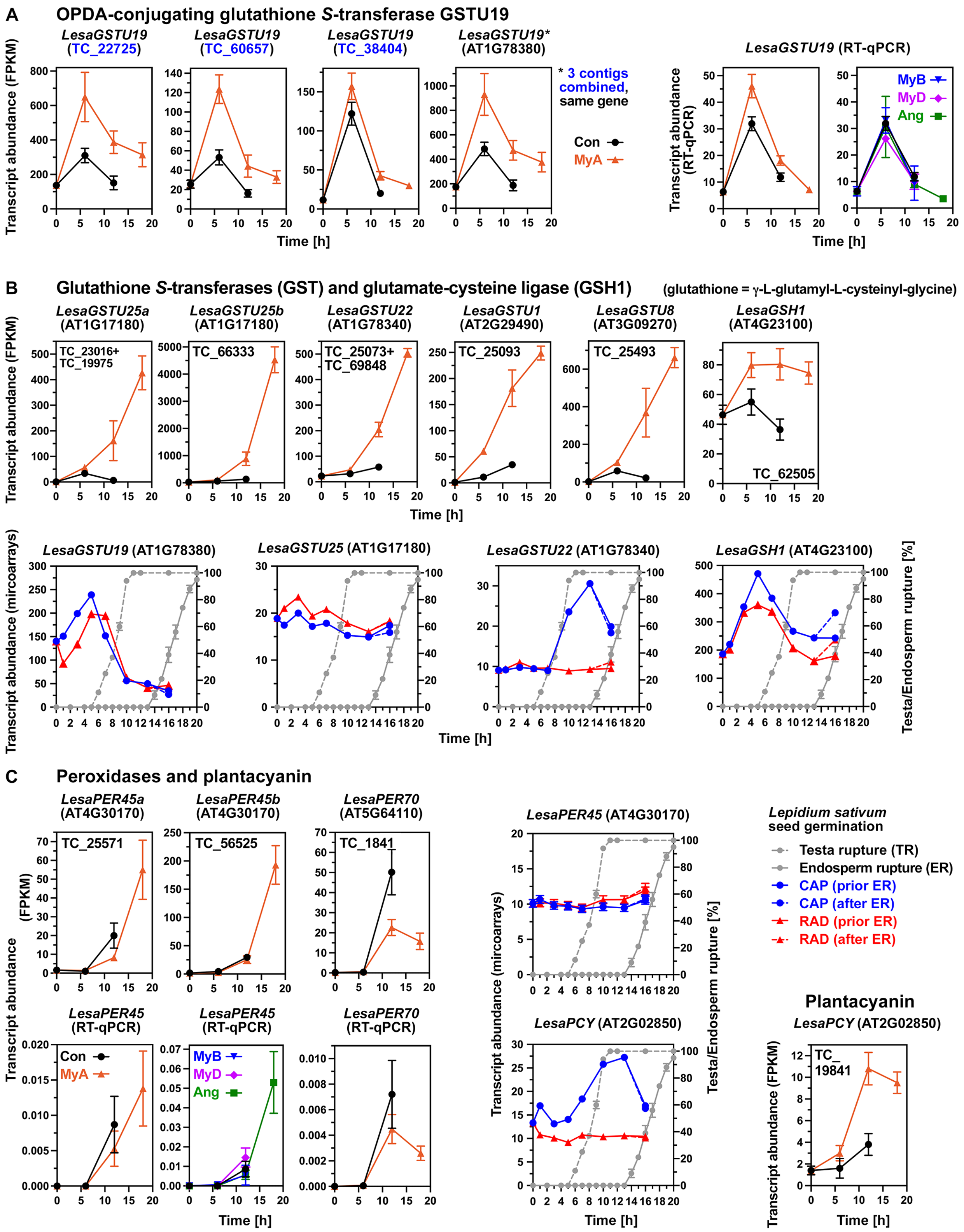

Figure S7. The effect of myrigalone A (0.5 mM MyA) and other compounds on the expression patterns of glutathione *S*-transferase (GST) and peroxidase genes during *Lepidium sativum* (cress) seed germination. (**A**) Gene expression analyses for the OPDA-conjugating GSTU19 enzyme. Three distinct *LesaGSTU19* transcript contigs were obtained in the RNAseq analysis and based on their sequence comparison derived from the same gene. Their FPKM values were therefore combined to provide the combined transcript expression pattern of this *LesaGSTU19* gene. RT-qPCR was used to verify the expression pattern and the effects of MyA, MyB, MyD and angelicin (Ang). (**B**) The effect of MyA treatment (0.5 mM) on the transcript abundance patterns of GSTs and GSH1 in germinating

cress seeds. The names of *L. sativum* (*Lesa*) genes and the corresponding *A. thaliana* orthologs (AGI in brackets) are provided; see abbreviations for full names of genes. Mean ± SEM values (relevant transcript contigs (TC-IDs) included in each graph) are presented of 4-5 (FPKM) and 3 (qRT-PCR) biological replicates. Spatiotemporal expression pattern in the CAP and RAD compartments during cress seed germination derived from microarrays [40]. (**C**) The effect of MyA treatment (0.5 mM) on the transcript abundance patterns of peroxidases and plantacyanin in germinating cress seeds.

Figure S8

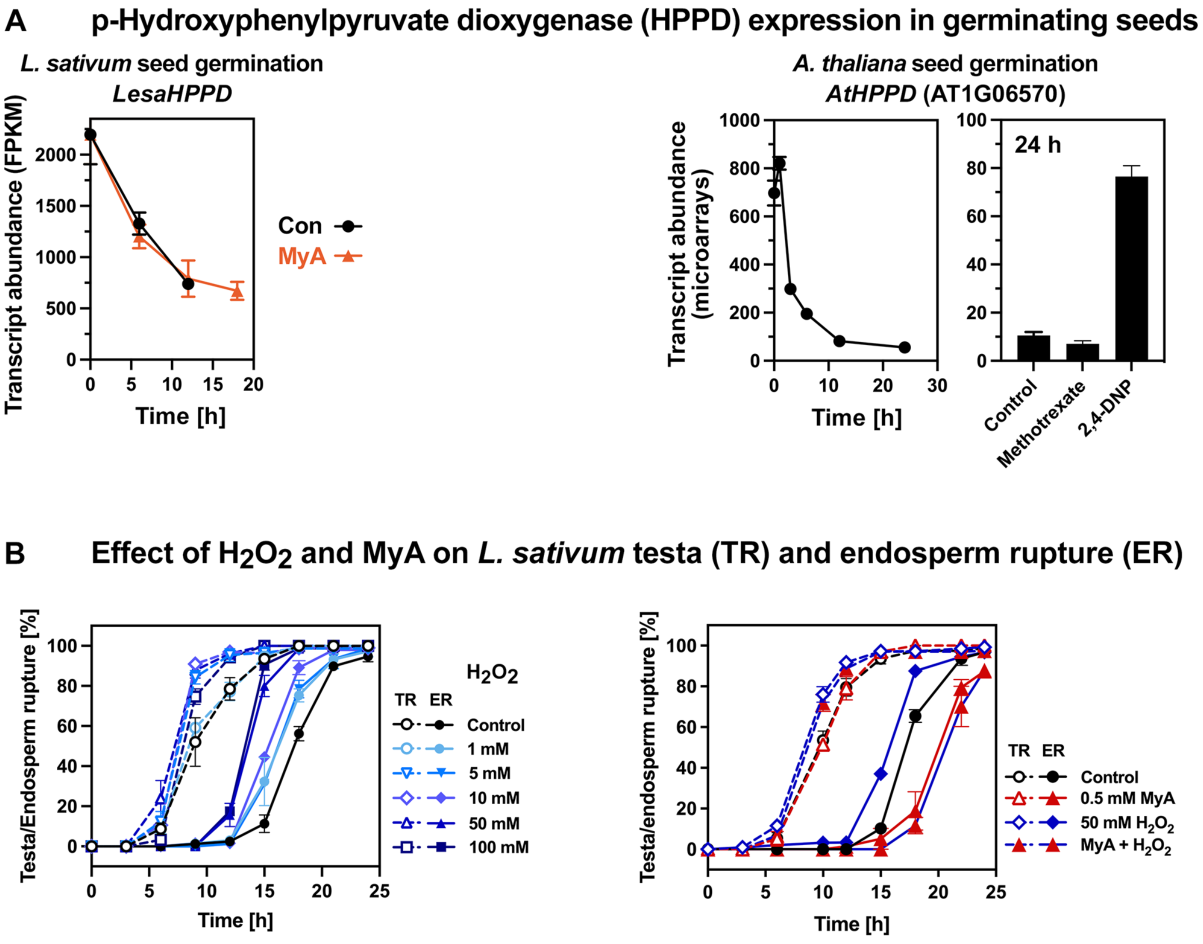

Figure S8. The effect of myrigalone A (0.5 mM MyA) and other compounds on the (**A**) expression of p-hydroxyphenylpyruvate dioxygenase (HPPD) during *Lepidium sativum* (cress) and *Arabidopsis thaliana* seed germination; the results for *A. thaliana* were derived from the work of Bassel et al [31] via the Arabidopsis eFP Browser at bar.utoronto.ca (Winter et al., 2007, PLoS One 2:e718). (**B**) The effects of H_2O_2 and MyA (0.5 mM) on the kinetics of testa rupture (TR) and endosperm rupture (ER) during cress seed germination. Seeds were incubated at 20 ºC in continuous white light, TR and ER scored over time, mean values ± SEM for 3 replicates each with ca. 30 seeds are shown.

Figure S9

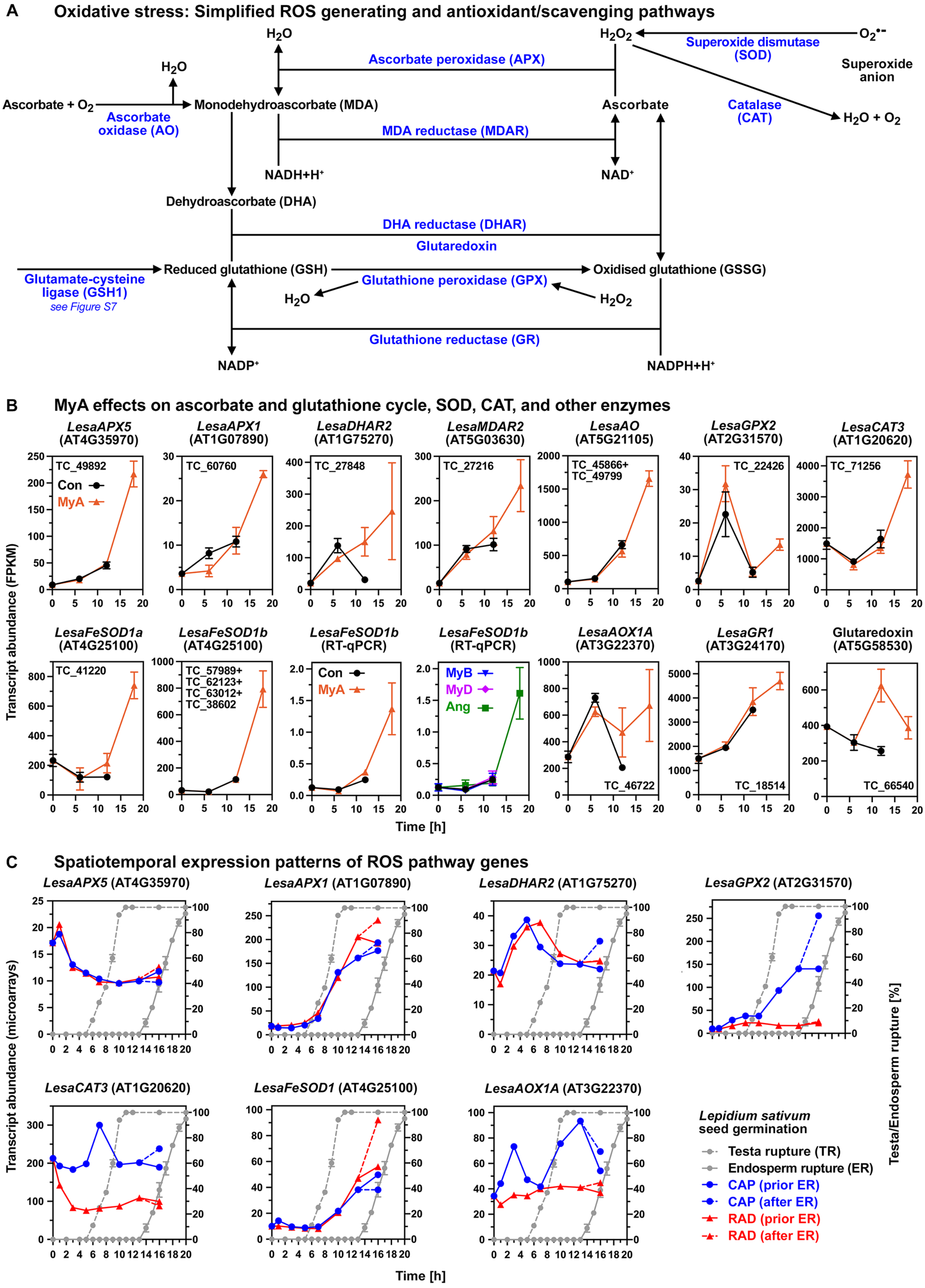

Figure S9. The effect of myrigalone A (0.5 mM MyA) and other compounds on the expression patterns of ascorbate and glutathione cycle, and other genes involved in scavenging pathways for reactive oxygen species (ROS) during *Lepidium sativum* (cress) seed germination. (**A**) Simplified

scheme of ascorbate and glutatione scavenging pathways. (**B**) The effect of MyA on the transcript abundance patterns in germinating cress seeds. The names of *L. sativum* (*Lesa*) genes and the corresponding *A. thaliana* orthologs (AGI in brackets) are provided; see abbreviations for full names of genes. Mean ± SEM values (relevant transcript contigs (TC-IDs) included in each graph) are presented of 4-5 (FPKM) and 3 (qRT-PCR) biological replicates. (**C**) Spatiotemporal expression patterns in the CAP and RAD compartments during cress seed germination derived from microarrays [40].

Figure S10

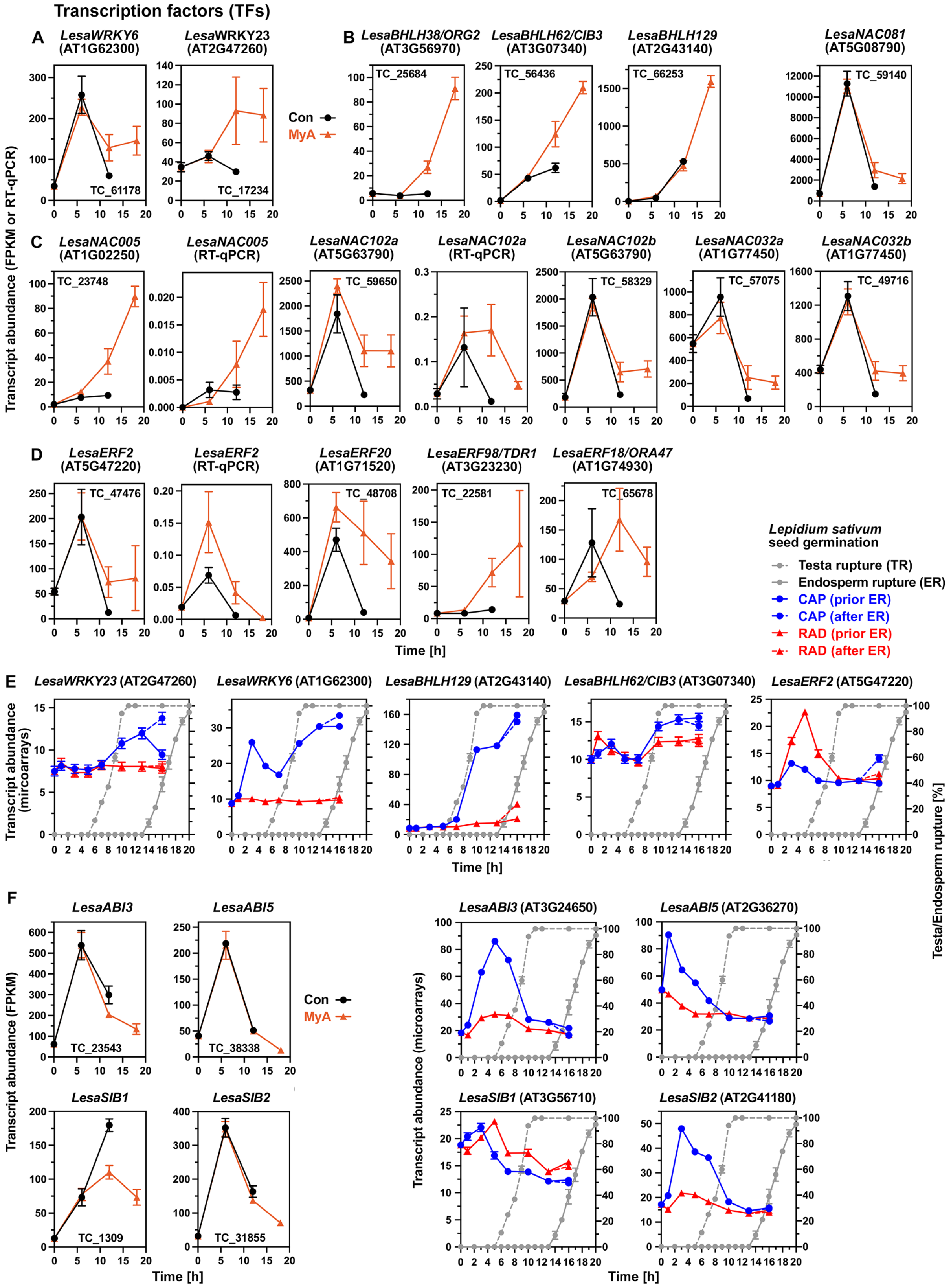

Figure S10. The effect of myrigalone A (0.5 mM MyA) on the expression patterns of transcription factor (TF) genes during *Lepidium sativum* (cress) seed germination. (**A-D**) The effect of MyA on the transcript abundance patterns in germinating cress seeds. (**E**) Spatiotemporal expression patterns in the CAP and RAD compartments during cress seed germination derived from microarrays [40]. (**F**) The effect of MyA and spatiotemporal expression patterns of the ABI3 and ABI5 TFs mediating

ABA-inhibition of seed germination, and of SIGMA FACTOR BINDING PROTEIN1 (SIB1) and SIB2 which physically interact with the WRKY75 TF to inhibit its activity in seed germination [61]. Mean ± SEM values are presented, for details see Figure S9.

Figure S11

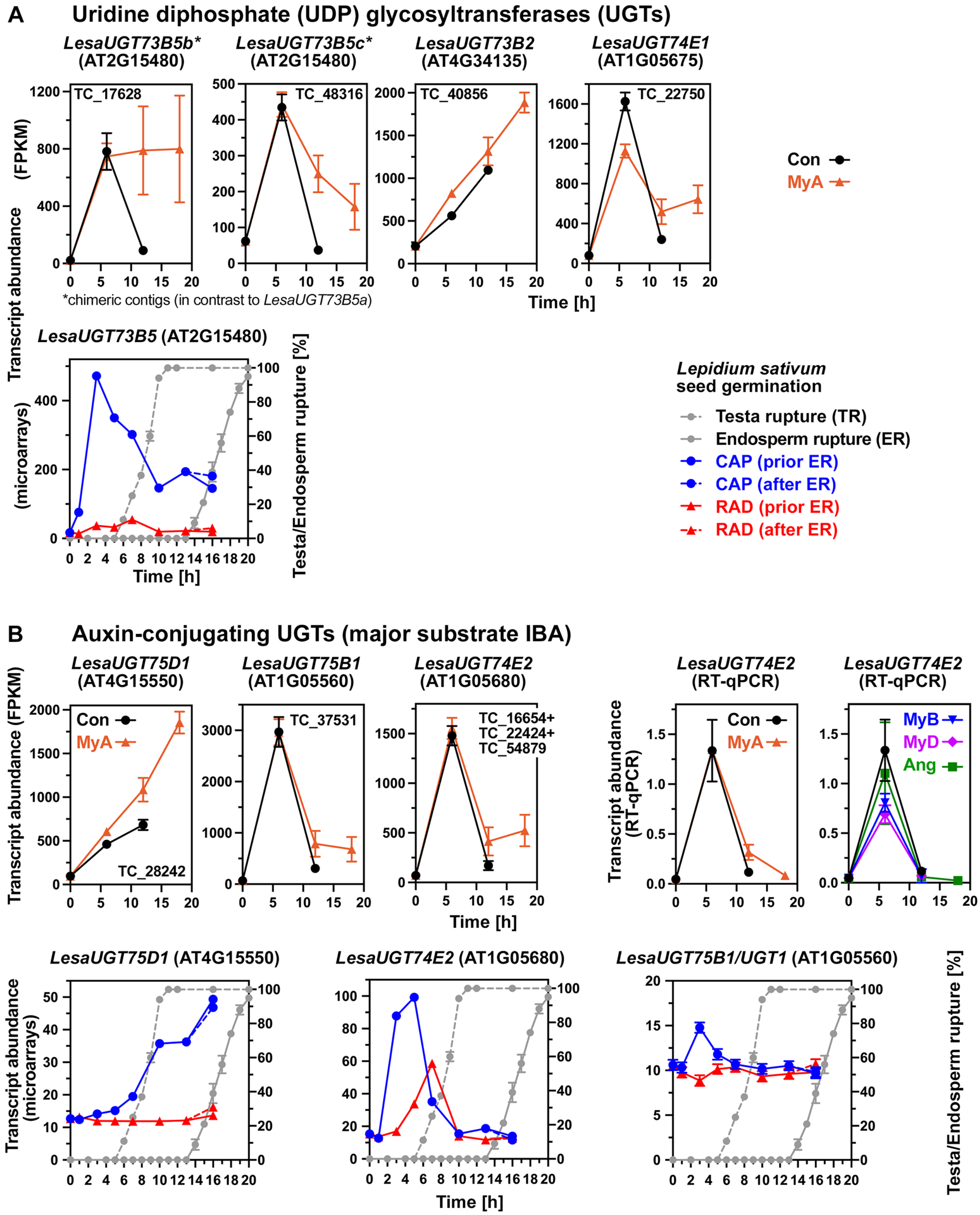

Figure S11. The effect of myrigalone A (0.5 mM MyA) on the expression patterns of UDP glycosyltransferase (UGT) genes during *Lepidium sativum* seed germination. Mean ± SEM values are presented, for details see Figure S9.

Figure S12

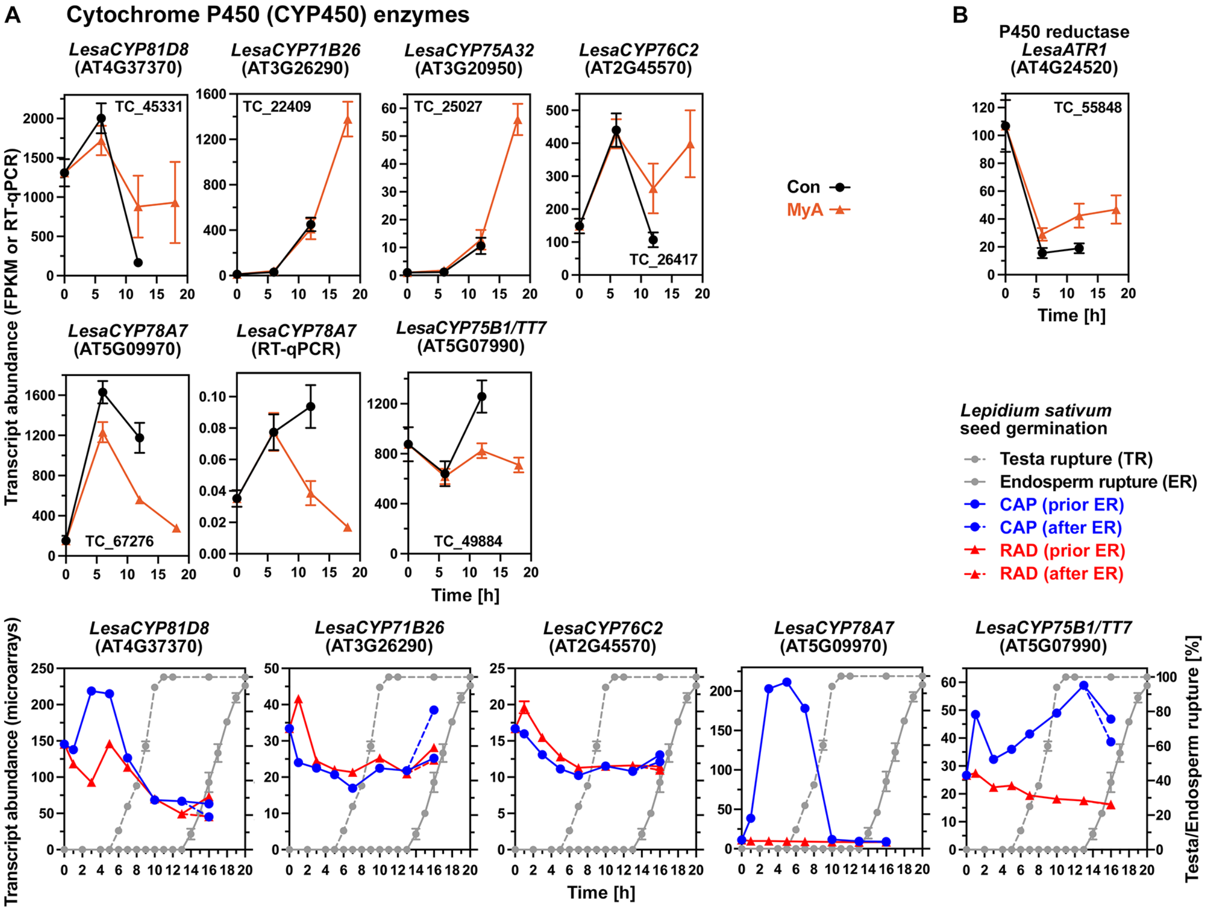

Figure S12. The effect of myrigalone A (0.5 mM MyA) on the expression patterns of (**A**) cytochrome P450 (CYP450) and (**B**) P450 reductase genes during *Lepidium sativum* seed germination. Mean ± SEM values are presented, for details see Figure S9.

Figure S13

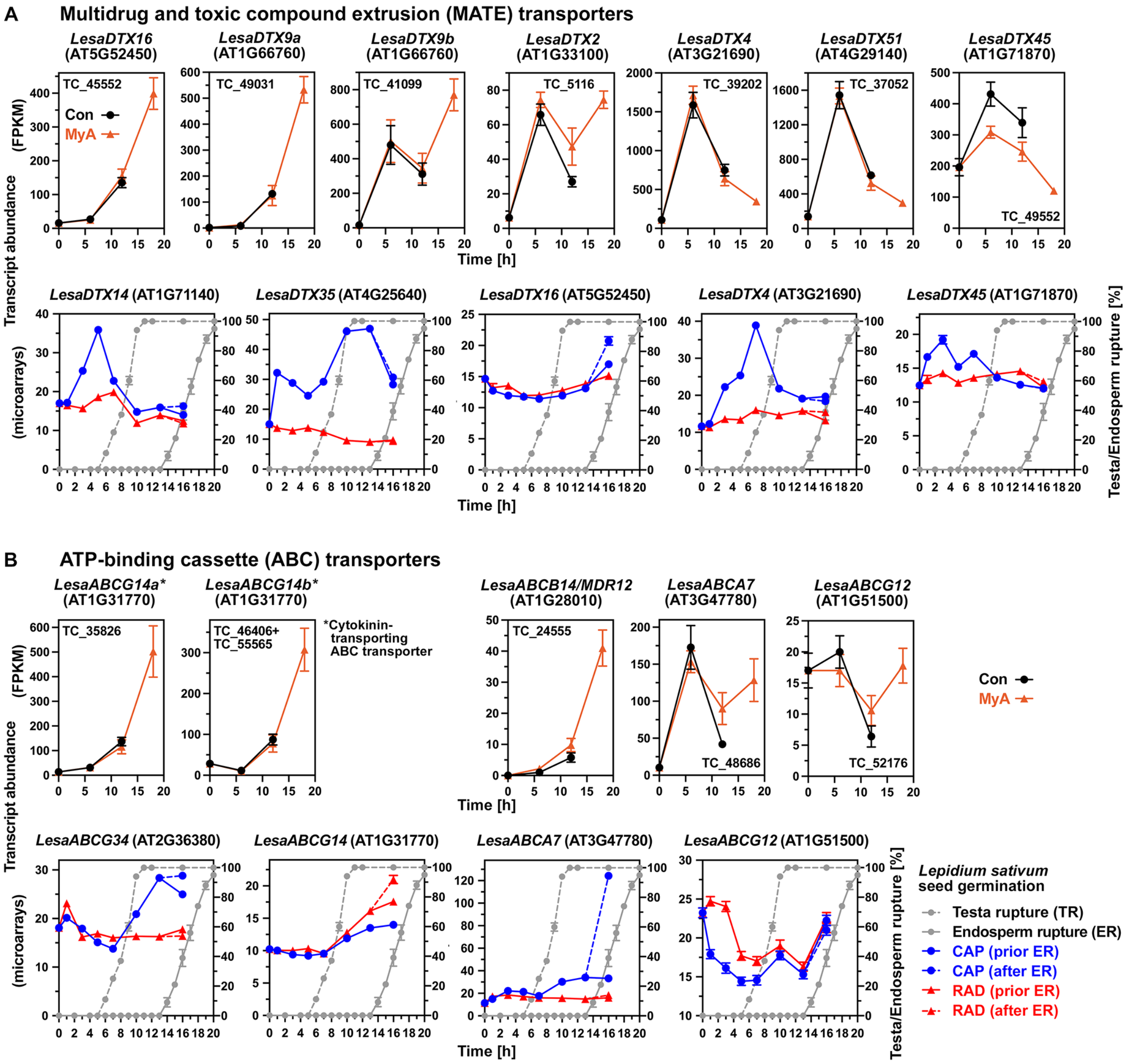

Figure S13. The effect of myrigalone A (0.5 mM MyA) on the expression patterns of (**A**) MATE and (**B**) ABC transporter genes during *Lepidium sativum* seed germination. Mean ± SEM values are presented, for details see Figure S9.

Figure S14

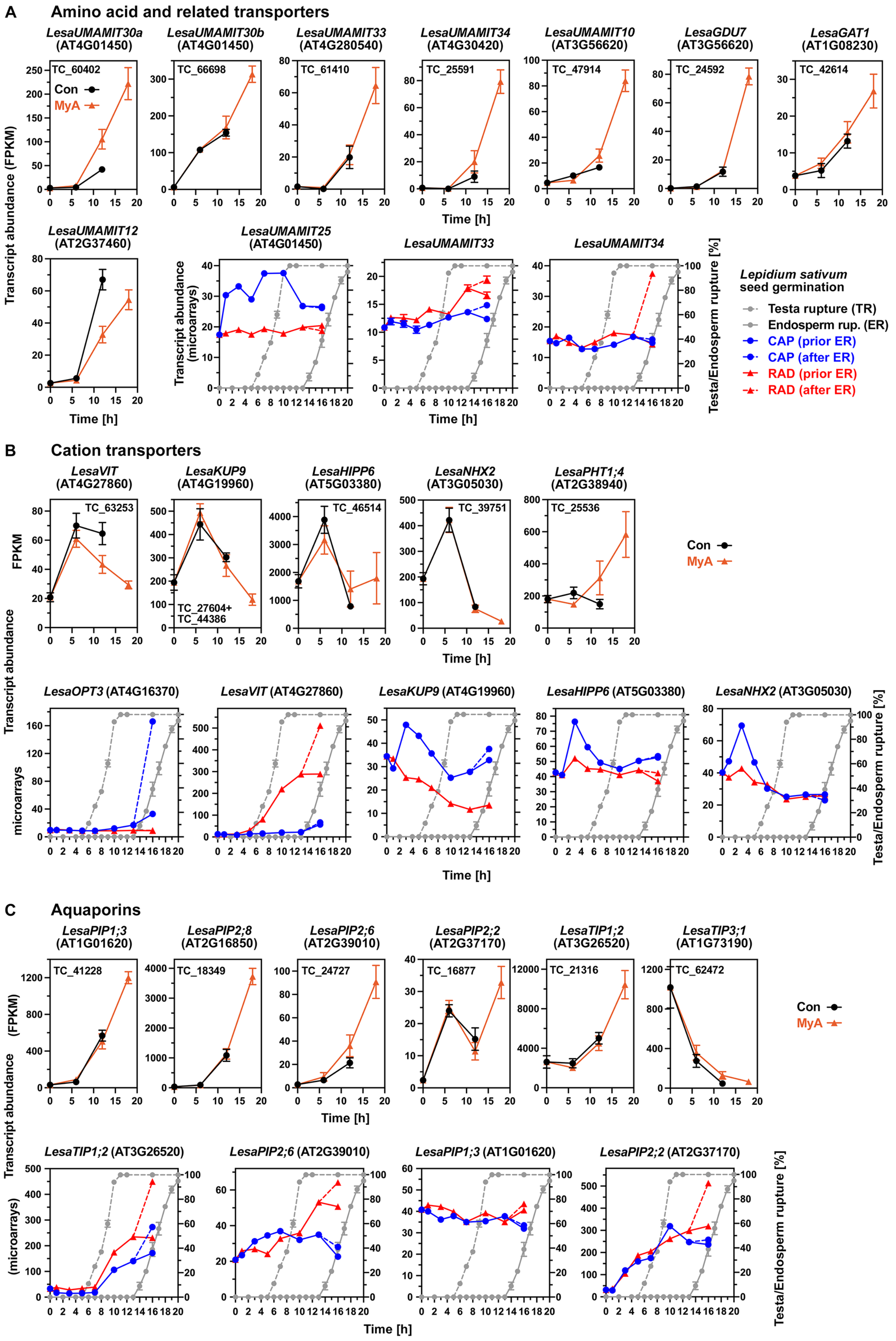

A Amino acid and related transporters
LesaUMAMIT30a (AT4G01450)
LesaUMAMIT30b (AT4G01450)
LesaUMAMIT33 (AT4G280540)
LesaUMAMIT34 (AT4G30420)
LesaUMAMIT10 (AT3G56620)
LesaGDU7 (AT3G56620)
LesaGAT1 (AT1G08230)
TC_60402
TC_66698
TC_61410
TC_25591
TC_47914
TC_24592
TC_42614
Con
MyA
Transcript abundance (FPKM)
Time [h]
LesaUMAMIT12 (AT2G37460)
LesaUMAMIT25 (AT4G01450)
LesaUMAMIT33
LesaUMAMIT34
Transcript abundance (microarrays)
Testa/Endosperm rupture [%]
Lepidium sativum seed germination
Testa rupture (TR)
Endosperm rup. (ER)
CAP (prior ER)
CAP (after ER)
RAD (prior ER)
RAD (after ER)
B Cation transporters
LesaVIT (AT4G27860)
LesaKUP9 (AT4G19960)
LesaHIPP6 (AT5G03380)
LesaNHX2 (AT3G05030)
LesaPHT1;4 (AT2G38940)
TC_63253
TC_27604+ TC_44386
TC_46514
TC_39751
TC_25536
FPKM
Transcript abundance
microarrays
LesaOPT3 (AT4G16370)
LesaVIT (AT4G27860)
LesaKUP9 (AT4G19960)
LesaHIPP6 (AT5G03380)
LesaNHX2 (AT3G05030)
C Aquaporins
LesaPIP1;3 (AT1G01620)
LesaPIP2;8 (AT2G16850)
LesaPIP2;6 (AT2G39010)
LesaPIP2;2 (AT2G37170)
LesaTIP1;2 (AT3G26520)
LesaTIP3;1 (AT1G73190)
TC_41228
TC_18349
TC_24727
TC_16877
TC_21316
TC_62472
Transcript abundance (FPKM)
(microarrays)
LesaTIP1;2 (AT3G26520)
LesaPIP2;6 (AT2G39010)
LesaPIP1;3 (AT1G01620)
LesaPIP2;2 (AT2G37170)

Figure S14. The effect of myrigalone A (0.5 mM MyA) on the expression patterns of (**A**) amino acid, (**B**) cation, and (**C**) aquaporin transporter genes during *Lepidium sativum* seed germination. Mean ± SEM values are presented, for details see Figure S9.

Figure S15

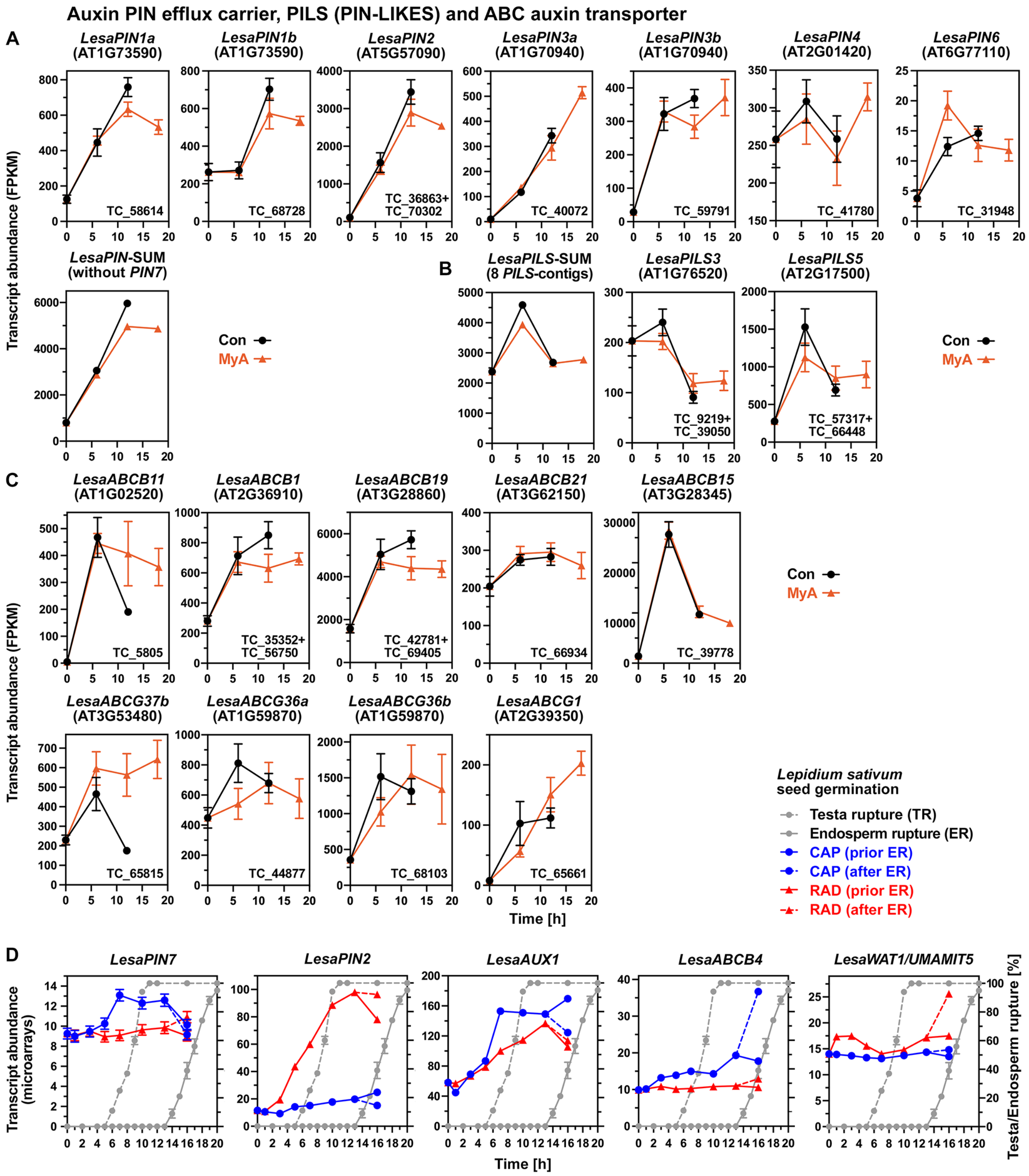

Figure S15. The effect of myrigalone A (0.5 mM MyA) on the expression patterns of auxin transporter genes during *Lepidium sativum* seed germination. (**A**) PIN (PIN-FORMED) IAA efflux carriers. (**B**) PILS (PIN-LIKES) auxin carrier. (**C**) Auxin-transporting ABC transporter genes. (**D**) Spatiotemporal expression patterns in the CAP and RAD compartments during cress seed germination derived from microarrays [40]. Mean ± SEM values are presented, for details see Figure S9.

Figure S16

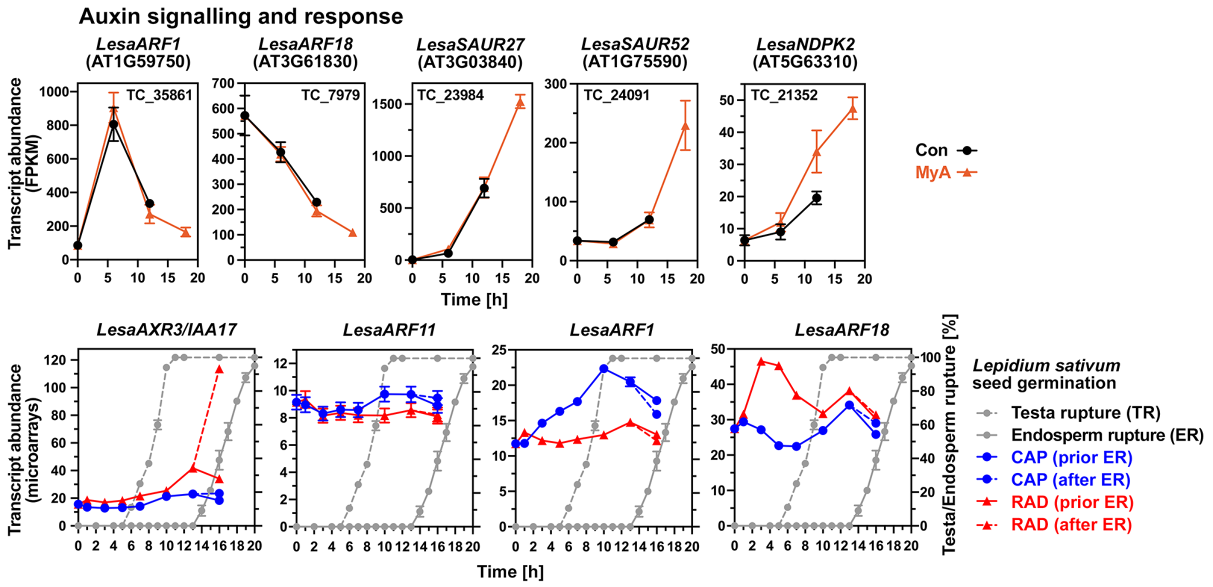

Figure S16. The effect of myrigalone A (0.5 mM MyA) on the expression patterns of auxin signalling genes during *Lepidium sativum* seed germination. Mean ± SEM values are presented, for details see Figure S9.

Figure S17

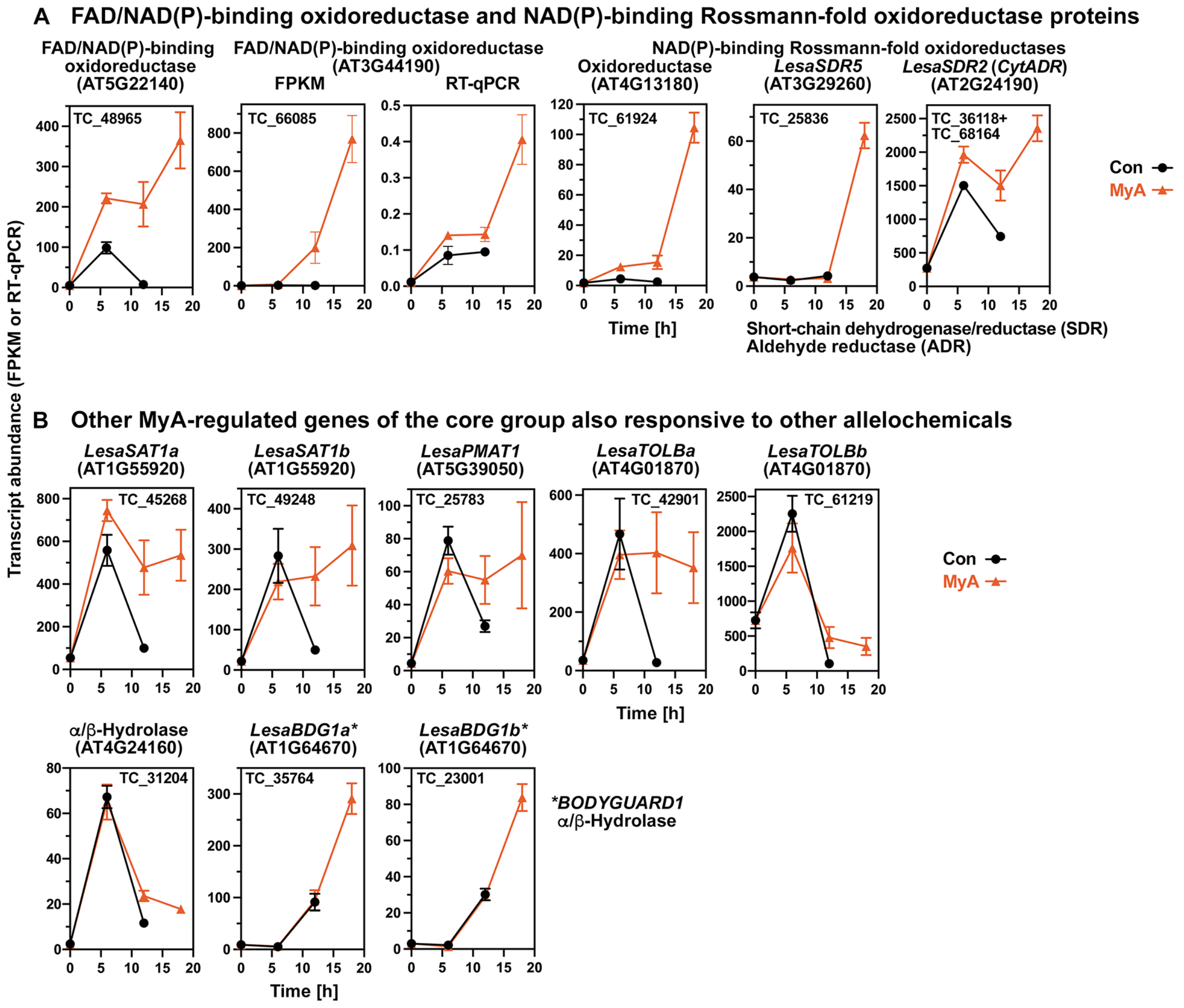

Figure S17. The effect of myrigalone A (0.5 mM MyA) on the expression patterns of (**A**) oxdioreductase and (**B**) other genes during *Lepidium sativum* seed germination. Mean ± SEM values are presented, for details see Figure S9.

Supplementary Table S1. Primer sequences used for RT-qPCR.

Gene name	Contig ID	Fw primer sequence (5'-3')	Rev primer sequence (5'-3')	Annealing Temp (ºC)	Amplicon size (bp)
**Target genes**					
ABCB4	TC_55627	TATCTTTCGTCGTCCTCGCG	TGCCTTGACGAATCCCTGTC	62	237
ABCG34	TC_67753	TTGCTCCTTCTGCTTCCAGG	ATCCATGGCGGGATGTCATC	62	199
ABCG37	TC_54077	TTTACTCCGCTTGGCTTCGT	TCTTGCGTCTAATCCCGTCG	62	236
AOX1A	TC_46722	GGAGGTAAGGCAGCGAGATC	GGCGAATCTCACACCTCCAA	64	183
ARF11	TC_70138	AAAGCACAATGCCTGCATCC	CTCCATCTTGAAGCTGGCCA	64	230
CYP78A7	TC_67276	TTAACGGAGTGGACCATGGC	AAGACTCCTTCACCACTGCG	64	154
CYP81D4	TC_3414	CGCTCCACCGAACATTCCTA	TAAAGTGAGGACGGTTCGCC	66	162
CYP81D8	TC_45331	TCAATCGCCGAAGAGTGCTT	GAGCTGCGATCATGCTTGTG	64	109
ERF2	TC_13835	AATAGCGGTGGTTCCAGCTC	CTGCATCTTCCGCTGTCTCA	62	149
FAD/NAD(P)-oxidoreductase	TC_66085	GTGGACCGAGATTGCTGGAA	AGGAAGTGACAATCTGCGTGT	64	181
FSD1	TC_62123	GGGGAAAGCATCACAGAGCT	AGCAGCGTTGTTGAAAGCAG	62	140
GSTU19	TC_60657	TTCTCGATTTCTGGCCGAGC	TGCTAGACCAAACCTCGTCG	66	222
GSTU25	TC_23016	TAGAGTCGGAGCTTGCAGAC	AGCCCAAGCAATCAGTTTTGG	60	155
LHT1	TC_67781	CCGGGTATTGCAGTCTTGGT	ACGATGCAAACGCCACATTC	62	197
monooxygenase	TC_38418	GGATCGGTGTTGTTCTTGCG	CAACTTTGACACCAGCACGG	66	141
NAC5	TC_23748	AAGAAGCTCAGCCGCAAGAT	ATGGCTGCAATTCTCCTGCA	62	119
NAC102	TC_59650	TTTCGGTTCCGGTTATCGCA	AGCACCAGTCGCTTTCCAAT	68	179
OPR1/2	TC_9428	TCCTCACACGCTAATGCCAA	AAGCCACAAGATCGGTTCGT	60	119
OXI1	TC_44356	GCAGCGGAACTTGTACTTGC	GGAGCGAGATTCGTGGAGAG	64	140
PER13	TC_24424	ATGACGAGAGGTTTGCTGCA	AGAGCAACGATGTCAGCACA	60	121
PER45	TC_56525	AGTGTCTCTGCTCAGCTTCG	ATCGACGCATCACATCCCTG	62	170
PER70	TC_1841	TCTGACCAGGTCCTGTGGAA	TTGGACATCGACCAAGCGAA	62	113
PIN7	TC_53214	CTCATCTACGCTCTGGTGGC	ATAGCCATCACTGCTGGTCC	60	215
SKS15	TC_26313	CAACAGTCGGACCAGCTCAT	AGTACCGTCCATGTGCCAAG	64	119
TAT2	TC_42921	CCGAGCTTCTGTACCGATCC	GGCTTGTGAACAACCAGCTG	66	195
UGT1	TC_37531	GTTCCAGTTGTGGCGTTTCC	CTTCTGCACCCGCTTCAAAC	60	223
UGT73B5	TC_66416	TTGCAGCAGGTCTACCGATG	GAACTTCCCTCACTGCCTCC	64	171
UGT74E2	TC_54879	CCACTGTCTTCCCCATGTCC	CCAAGGCTGCGTGAAAAACA	64	239
UGT75D1	TC_28242	ACAAGAAGCCATGACCTCGG	AGAACGACCTCCGACTCTGA	64	224
UMAMIT25	TC_24907	TCGCCATGTTTCTCGAACGA	GGACAGAACCATGGCTCCAA	60	287
WRKY23	TC_17234	AACGCAAACGCAGCTAATGG	GTTTGGAGTCGCTGGTGGAT	64	139
WRKY75	TC_64935	GAAGTGGGGCTGAGTCGAAG	ACGACGACTTCTTGGTCCAC	64	220
**Reference genes**					
PP2AA2	TC_69398	TTTTGCGTGCGGTTTCTCTG	CTGAGCTCCACAAGTCCAGG	60	202
CAC AP2M	TC_69491	GGAAATGAAATCGCGCCCAG	TTCCATGCGTGTACGACCAA	60	223
Hobbit	TC_38831	GGGGTTCGTGGGGAATCTTT	GTGCCTCCTGACACCTGTAC	60	232
